# The Association Between Menstrual Cycle Phase, Menstrual Irregularities, Contraceptive Use and Musculoskeletal Injury Among Female Athletes: A Scoping Review

**DOI:** 10.1007/s40279-024-02074-5

**Published:** 2024-08-31

**Authors:** Candice MacMillan, Benita Olivier, Carel Viljoen, Dina Christa Janse van Rensburg, Nicola Sewry

**Affiliations:** 1 Department of Physiology, Faculty of Health Sciences, University of Pretoria, South Street, Pretoria, 0083 Gauteng South Africa; 2 Sport, Exercise Medicine, and Lifestyle Institute (SEMLI), Faculty of Health Sciences, University of Pretoria, Pretoria, South Africa; 3 Centre for Healthy Living Research, Oxford Institute of Allied Health Research, Department of Sport, Health Sciences and Social Work, Oxford Brookes University, Oxford, UK; 4 Wits Cricket Research Hub for Science, Medicine and Rehabilitation, Department of Physiotherapy, School of Therapeutic Sciences, Faculty of Health Sciences, University of the Witwatersrand, Johannesburg, South Africa; 5 Department of Physiotherapy, Faculty of Health Sciences, University of Pretoria, Pretoria, South Africa; 6 Section Sports Medicine, Faculty of Health Sciences, University of Pretoria, Pretoria, South Africa; 7 International Olympic Committee (IOC) Research Centre, Pretoria, South Africa

## Abstract

**Background:**

The influence of menstrual cycle phases (MCPs), menstrual irregularities (MI) and hormonal contraceptive (HC) use on injury among female athletes has been scrutinised. Existing systematic reviews investigating the effect of exposures affecting the endogenous reproductive hormone status on sporting injuries are limited in terms of the types of studies included and injuries investigated.

**Objective:**

This scoping review aims to summarise the coverage of the literature related to the extent, nature and characteristics of the influence of MCP, MI and HC use on musculoskeletal injuries among athletes. It also aims to summarise key concepts and definitions in the relevant literature. Observational and experimental studies investigating the effect of MCP, MI, and HC on musculoskeletal injuries among female individuals of reproductive age were included. Studies specifically stating pregnant women, perimenopausal/postmenopausal athletes, or those using medication (other than HC) that affects reproductive hormone profiles or the musculoskeletal system were excluded.

**Methods:**

This scoping review was conducted according to the Preferred Reporting Items for Systematic Reviews and Meta-Analyses Extension for Scoping reviews and JBI scoping review guidelines. Published and unpublished studies were sourced from several databases and resources. Initial keywords used included terms related to “menstrual cycle”, “hormonal contraception” and “injury.” Titles and abstracts of identified citations were screened independently and assessed for eligibility by two independent reviewers. Data from the included studies were extracted using a standard data extraction form.

**Results:**

The search yielded 10,696 articles, of which 96 met the eligibility criteria. Most studies investigated MI (77%), and 49% included MCP as a contributing injury risk factor. Publications have increased over the last two decades. Collectively, only 16% of research has been conducted in Africa, Asia and Oceania. There were no studies from South America. Seventy-five percent of the studies investigated individual versus team (25%) sport athletes. Most studies only investigated elite or professional (*n* = 24; 25%) level athletes. The definitions of injury, eumenorrhea and MI differ vastly among studies. Regarding MI, most studies (69%) investigated secondary amenorrhea, followed by oligomenorrhea (51%) and primary amenorrhea (43%). Concerning HC, the influence of oral contraceptive pills was mainly investigated.

**Conclusions:**

Research related to MCP, MI and HC as contributing musculoskeletal injury risk factors is increasing; however, several gaps have been identified, including research from countries other than North America and Europe, the study population being non-professional/elite level athletes, athletes participating in team sports and specific injuries related to MCP, MI and HC, respectively. Differences in methodology and terminology of injury, MCP and MI hinder comparative summative research, and future research should consider current published guidelines during the study design. Identifying barriers to following standard guidelines or research investigating the most practical yet accurate methods to investigate the influence of MCP on musculoskeletal health might yield valuable insights for future research designs.

**Clinical Trial Registration:**

Scoping review registration number: Open Science Framework (10.17605/OSF.IO/5GWBV).

**Supplementary Information:**

The online version contains supplementary material available at 10.1007/s40279-024-02074-5.

## Key Points

**Table:** 

Research on menstrual cycle phases, menstrual irregularities and hormonal contraceptives as musculoskeletal injury risk factors is increasing, but gaps remain, especially outside North America and Europe.
Methodological and terminological differences hinder comparative research, emphasising the need for standardised guidelines.
Identifying barriers to following guidelines and practical research methods can improve future studies on the impact of menstrual cycle phases on musculoskeletal health.

## Introduction

Injury rates in gender-comparable sports are higher among female athletes participating at different levels of play [1–3]. Acknowledgement of gender-based differences in injury presentation is necessary for holistic injury prevention, assessment, and treatment to allow for a successful return to play and a reduced risk of re-injury [4, 5]. The influence of the menstrual cycle phases (MCPs) on injury among female athletes has been a subject of scrutiny [6–8]. Continuous hormonal fluctuations throughout the MCP seem to affect the material structure and mechanical properties of muscle [3], tendon [2], bone [6] and ligaments [1, 9]. The use of hormonal contraceptives (HCs) and menstrual irregularities (MI) influences the fluctuation of reproductive hormones on athletes’ injury risk and it therefore, also warrants investigation [5, 6].

### Menstrual Cycle Phases (MCPs) and Injury Risk

A normal MCP consists of the follicular phase that precedes ovulation, followed by the luteal phase [10–12]. The fluctuation of reproductive hormones throughout the MCP is associated with an increased risk for acute [1, 9, 13, 14] and overuse [2, 3, 15] injuries. Female footballers’ injury incidence rates are greater in the late follicular phase (47%) compared with the early follicular and luteal phases (32%) [14]. Studies also suggest an increased risk of muscle and tendon injuries [3, 14], as well as anterior cruciate ligament (ACL) injuries [9] during the late follicular phase compared with the luteal phase. A recent systematic review [16] suggests that peak oestradiol in the ovulatory phase is associated with laxity, strength and poor neuromuscular control, which, in turn, can predispose athletes to injury. Data have, however, not been consistent, and a direct causal relationship to injury has not yet been established.

### Menstrual Irregularity (MI) and Injury Risk

Menstrual irregularity is characterised by oligomenorrhoea, polymenorrhoea, amenorrhoea, anovulatory or luteal-phase deficient cycles [6, 8, 17] and is prevalent among athletes participating in different sports and is associated with an increased injury risk [6, 17]. High school athletes with MI sustain more severe injuries in terms of time loss than athletes with regular menses [17]. The female athlete triad (FAT) refers to the interrelatedness of MI, low energy availability and diminished bone mineral density [6, 17]. More recently, the FAT is called relative energy deficiency syndrome (RED-S); a condition resulting from insufficient energy intake relative to the energy expended, leading to impaired physiological functioning, including but not limited to metabolic rate, menstrual function, bone health, immunity and cardiovascular health. It is crucial for both male and female athletes, particularly emphasising the significant health and performance implications for female athletes [18, 19]. Several studies have investigated the association between MI and bone injuries and found that adolescent [13, 17] and adult [6] athletes with current or past menstrual dysfunction are particularly prone to bone stress injuries.

### Hormonal Contraceptive (HC) Use and Injury Risk

Hormonal contraceptives are used by approximately half of female athletes [20]. There are many types of HCs including oral contraceptive pills (OCPs), intrauterine devices (IUDs), injections, transdermal patches, implants and vaginal rings [20]. Exogenous hormones inhibit endogenous hormone production, eliminating normal hormonal fluctuations throughout the normal menstrual cycle [6, 21]. Considering the effects of natural MCP hormonal fluctuations on injury risk, several studies propose that the use of OCPs may, therefore, be protective against injury [1]*.* Among the general population, combined HCs are not protective against musculoskeletal (MSK) injuries [22]. Among female athletes, using OCPs in combination with [1] or without [23] neuromuscular training may increase the dynamic stability of the knee joint and decrease ACL injury risk.

A preliminary search of MEDLINE, the Cochrane Database of Systematic Reviews and JBI Evidence Synthesis was conducted. Systematic reviews investigating the effects of the MCP on athletes’ performance [24], ACL injuries [1, 8, 9] and tendinopathies [1, 9] have been published. The most recent review [16] exploring the MCP and sports injuries only included eight studies. One review appraised the literature regarding the association between combined HC and MSK injuries and conditions but included all female individuals and not only female athletes [22]. True to their nature, systematic reviews delve into the particulars of evidence related to the specific topic, and the literature summarised and appraised is often limited by stringent eligibility criteria [25].

As the number of female athletes grows, the number of publications related to female-specific injury risk factors is increasing. While systematic reviews on the effect of MCP, MI and HC use have been published, studies on these topics that are not included because of minor deviations from the eligibility criteria might be overlooked. Therefore, the primary aim of this scoping review is to determine the scope of the literature related to the association between MCP, MI and HC use and MSK injury among female athletes and identify gaps to aid the planning and commissioning of future research. The heterogeneity in the methodology and definitions of MCPs and MIs has been highlighted as a limitation to conducting meta-analyses and comparing findings [22, 26, 27]. An additional aim of this scoping review is to summarise key concepts and definitions in the relevant literature. A scoping review was deemed most appropriate for these outcomes because it is exploratory and incorporates a variety of research designs, focusing on coverage rather than the depth of a topic.

## Review Questions

The following broad research questions were proposed following preliminary literature searches and multidisciplinary discussions within the group.

- What research is available regarding the association between MCP, MI and HC use on MSK injuries among female athletes?
- How are MCP, MIs and injuries defined in research investigating the association between MCP, MI and HC use on MSK injuries among female athletes?
- Which methods are used to confirm different MCPs in research investigating the MC–injury association?
- Which types of MIs and HCs have been included in research investigating the influence of MI on injury risk?
- What are the evidence gaps in these fields?

## Eligibility Criteria

A detailed description of the eligibility criteria is summarised in Table 1 of the Electronic Supplementary Material (ESM) according to population, concept, context and study design. In short, studies investigating the effect of MCP, MI and HC on MSK sporting injuries among female athletes were included. Female athletes [28] and exercisers [29], participating in structured individual or team sports, regardless of the level of participation, who are of reproductive age, were considered. All menstruating women, regardless of regularity, and HC users (regardless of form) were included. Pregnant, perimenopausal and menopausal women or women using medications (other than HCs) that affect reproductive hormone profiles or the MSK system were excluded. All observational and experimental studies were considered. Studies published in languages other than English were not considered.

## Methods

This review was conducted according to the Preferred Reporting Items for Systematic Reviews and Meta-analyses Extension for Scoping Reviews (PRISMA-ScR) [30] and JBI guidelines [31] and registered on the Open Science Framework (identifier: 10.17605/OSF.IO/5GWBV).

### Search Strategy and Information Sources

The search strategy was developed with input from CM, NS and BO using the Peer Review of Electronic Search Strategies standard [32]. In addition, the search strategy was peer reviewed by a research librarian with expertise in systematic review searching and is not otherwise associated with the project [32]. A sequential three-step search strategy was utilised to find both published and unpublished studies before 30 November, 2023. First, a restricted search of the MEDLINE and Cumulative Index to Nursing and Allied Health Literature (CINAHL) databases was conducted, followed by an analysis of the text words contained in the title and abstract and of the index terms used to describe the article. Thereafter, a second search was undertaken across all the identified keywords and index terms across all included databases. Last, the reference lists of all identified studies was perused to identify any other relevant studies. Studies published in English were considered.

**Fig. 1:**
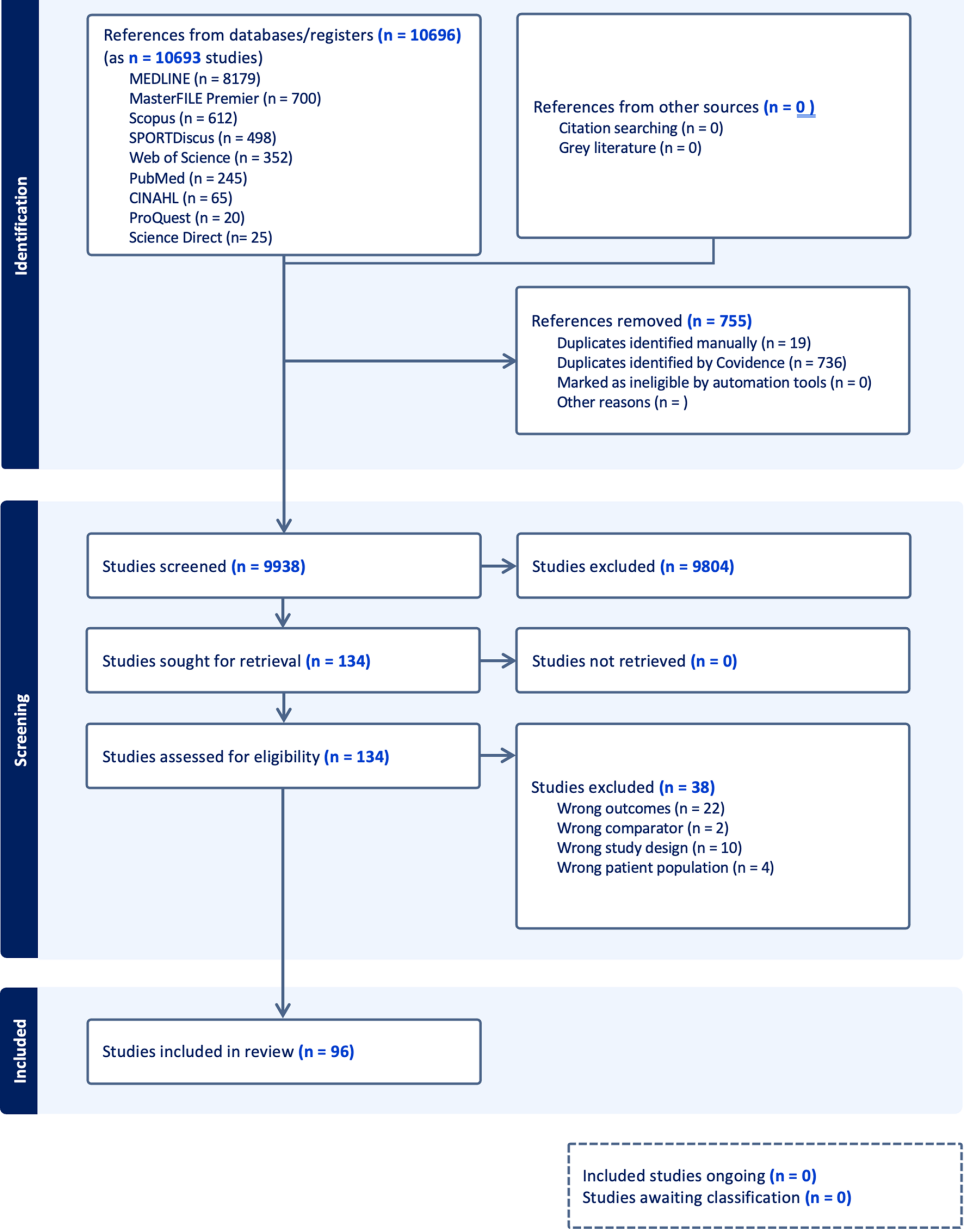
Preferred reporting items for systematic reviews and meta-analyses extension for scoping reviews (PRISMA-ScR) flow diagram

Databases searched included MEDLINE via PubMed, CINAHL, the Cochrane Controlled Trials Register in the Cochrane Library, Physiotherapy Evidence Database (PEDro), ProQuest 5000 International, ProQuest Health and Medical Complete, EBSCO MegaFile Premier, Science Direct, SPORTDiscus with Full Text and SCOPUS. The search for unpublished studies included EBSCO Masterfile Premier, OpenGrey (SIGLE), Worldwidescience.org, Google Scholar, Mednar and WiredSpace. Initial keywords used were terms related to “menstrual cycle”, “contraception” and “injury”. The filter function “humans” was applied where possible. A full search strategy for the PubMed database is detailed in Fig. 1 of the ESM. The one with the longest follow-up was selected for studies with multiple publications of the same outcome(s) reported. If older publications refer to articles, those included were accessed to clarify methods where necessary. When in doubt regarding a study’s eligibility criteria, the reviewers contacted the authors with a maximum of three attempts, two e-mails and one phone call (if possible), over 4 weeks.

### Sources of Evidence

All search results were uploaded and stored in a systematic review management platform (Covidence systematic review software; Veritas Health Innovation, Melbourne, VIC, Australia) and were accessible to all reviewers. Covidence automatically removed duplicates by reviewing the following fields: titles, year, volume and authorship. Two reviewers (CM and NS) independently checked the duplicates removed by Covidence and verified their accuracy. Two independent reviewers (CM and NS) screened all records’ titles, abstracts and full texts (where indicated) for inclusion. Reasons for excluding sources of evidence in the full text that did not meet the inclusion criteria were recorded and reported in the scoping review. Any disagreements with the reviewers were resolved through discussion or the use of a third reviewer (BO). All three reviewers have previous experience in evidence synthesis.

### Data Extraction and Charting Process

Four independent reviewers (CM, NS, BO and CV) extracted data from papers included in the scoping review. The data extracted included specific details about the study characteristics (i.e. design, location, study aim), (b) participant characteristics (i.e. eligibility criteria, age, training status), (c) context characteristics (i.e. type of sport, level of participation), (d) exposure characteristics (i.e. MCP, definitions and methods of determining participants’ MCP, type of MI, type, dosage and duration of HC use) and (e) injury incidence and characteristics (injury mechanism, body area/structure injured, type of the injury, primary or recurrent injury, injury definition) and key findings relevant to the review questions. Any reviewer disagreements were resolved through discussion or with an additional reviewer (CJR). Where appropriate, authors of papers were contacted to request missing or additional data.

### Data Analysis and Presentation

The results are presented numerically and thematically. A numerical analysis maps the data in a tabular and diagrammatic form, showing the distribution of studies by theme (MCP, MI, HC use or a combination of MCP, MI and/or HC), publication period, country of origin and study method. The charting of the results was based on an iterative approach. A descriptive analysis pertaining to themes and key concepts relevant to the research questions accompanies the tabulated results and describes how the results relate to the review objective and questions.

## Results

### Search Results

An initial search was performed in December 2022 and updated in December 2023. A total of 10,696 articles were found. After duplicate removal and screening, 96 articles (listed in Tables 2a–2g of the ESM) met the eligibility criteria and were included in this scoping review. The search results and the study inclusion process are reported in full in the PRISMA-ScR flow diagram (Fig. 1) [30].

### Themes

**Fig. 2:**
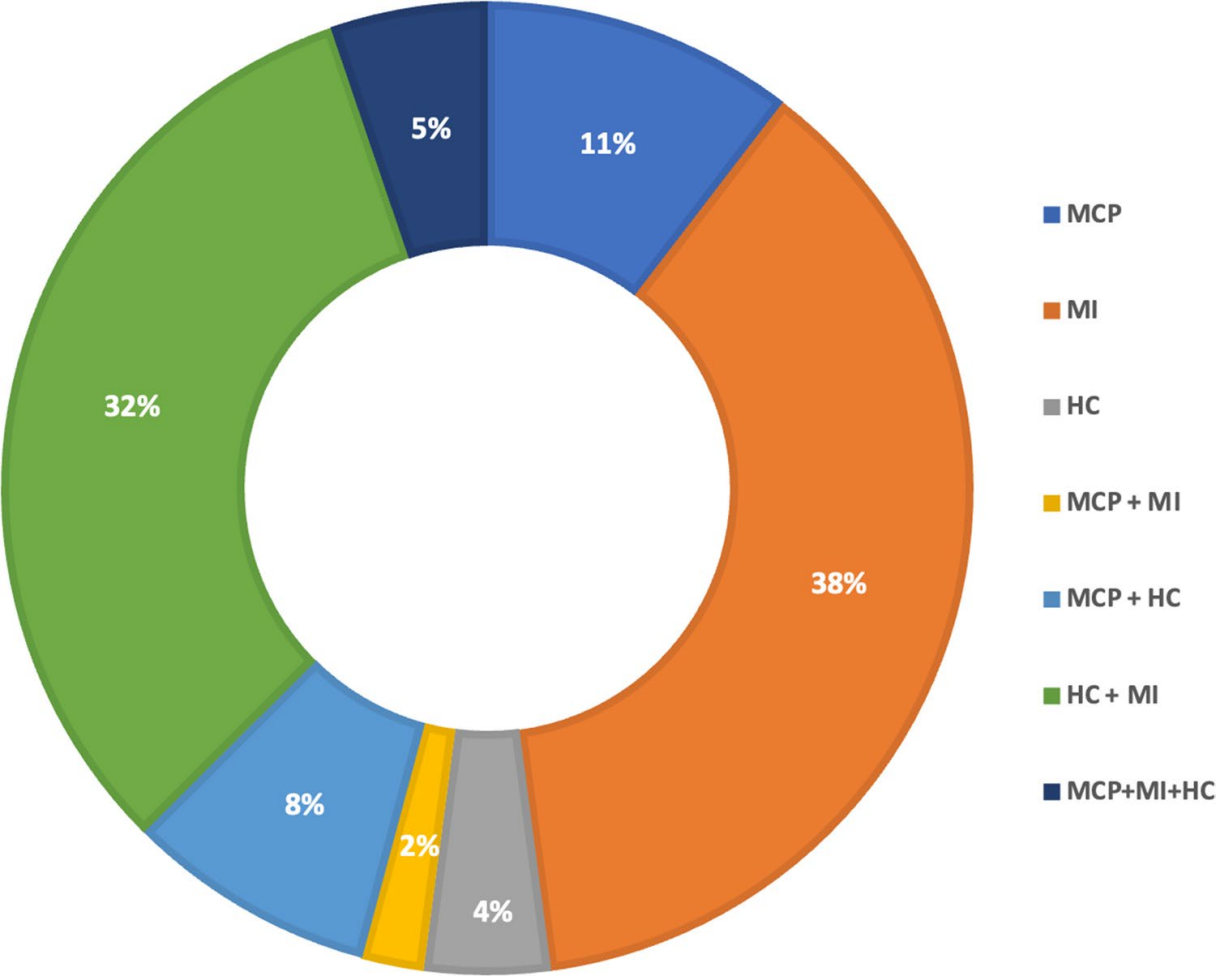
Percentage of studies investigating the relationship between injury and menstrual cycle phase (MCP), menstrual irregularity (MI) and hormonal contraceptives (HC), respectively or combined

Figure 2 depicts the number of studies investigating the association between MSK injury and MCP, MI or HC, respectively, and those investigating two or all of the themes. Although summarising the findings of the studies was not the primary purpose of this review, a summary of the aims and findings of each included study is included in Tables 2a–2g of the ESM. The high number of citations associated with some statements in the results section may reduce the reader’s experience, therefore, references to studies referred to in the results section are available in Tables 2a–2g of the ESM.

### Study Characteristics

#### Study Design

Study design by count and frequency were cross-sectional (43/96;45%), cohort (21/96;22%), case control (12/96;14%), case series (11/96;11%), case study (7/96;7%) and randomised controlled trial (1/96;1%).

#### Year of Publication of Articles Included

**Fig. 3:**
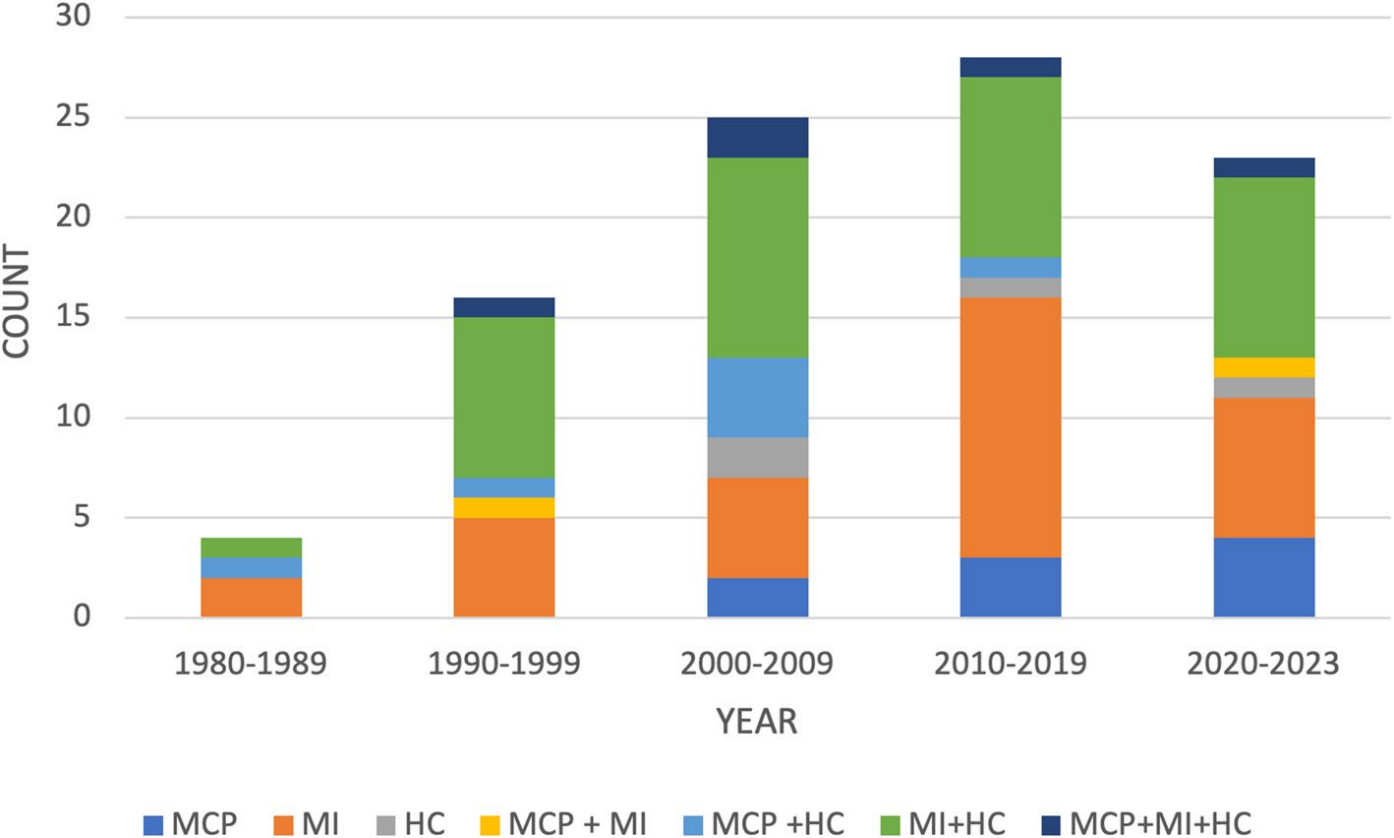
Year of publication reported by theme and decade. Themes: menstrual cycle phase (MCP); menstrual irregularity (MI); and hormonal contraceptive (HC)

The year of publication of the included publications ranged from 1986 to 2023. Figure 3 illustrates the distribution of publications per decade since 1980. Publications increased over time, with most (*n* = 28; 29%) published between 2010 and 2019. Notably, 23 (24%) publications have been published in the last 4 years. Furthermore, the number of publications related to the MCP as an MSK injury risk factor is increasing.

#### Geographical Location of Included Articles

**Fig. 4:**
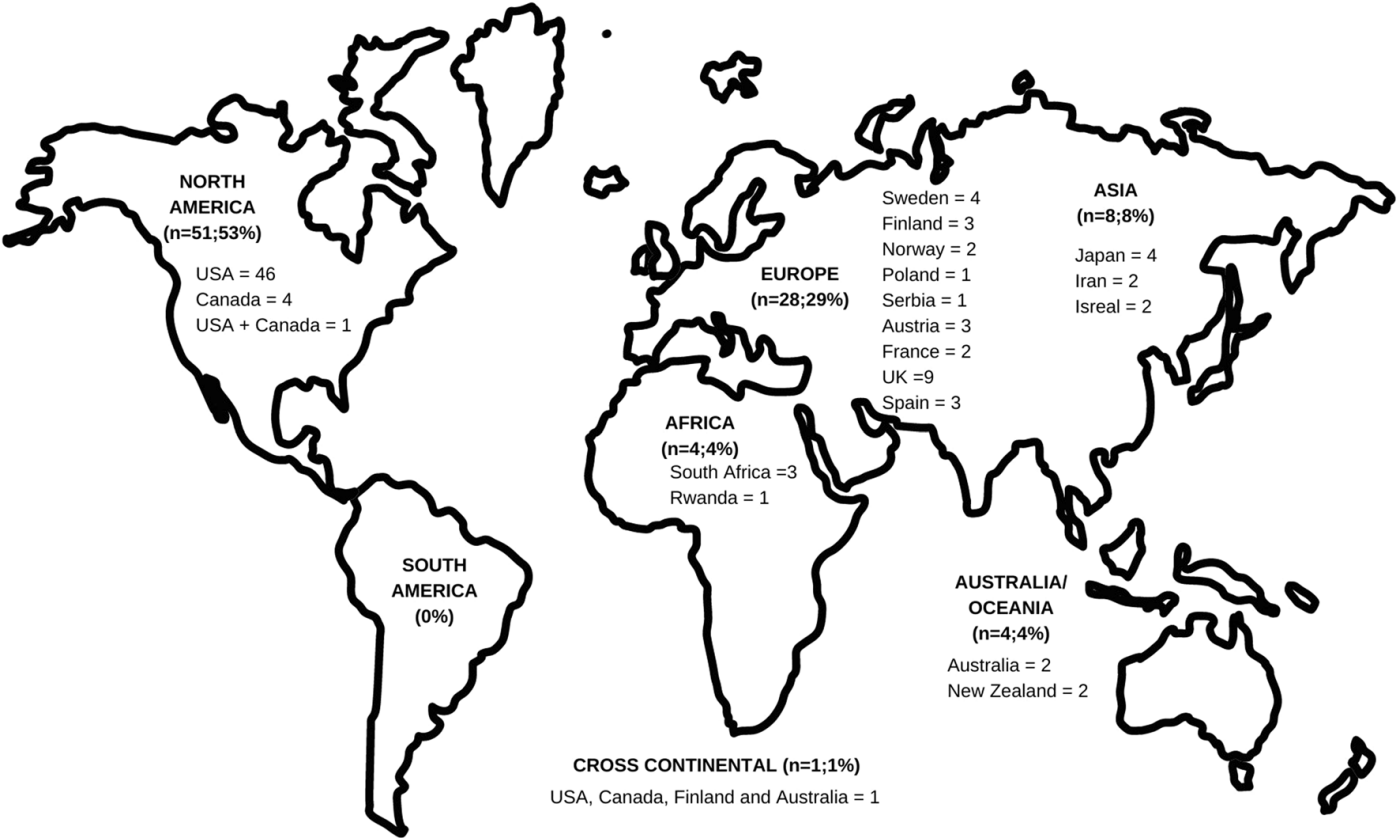
Geographic distribution of studies included by continent (*n*;%) and country (*n*)

Figure 4 demonstrates the distribution of studies included by continent and country. Most studies’ data collections (*n* = 51; 53%) were performed in North America, specifically the USA (*n* = 46; 43%), while no research emerged from South America. Only two studies included participants from more than one country [33], one of which was conducted across continents [34].

#### Sports Investigated

Tables 2a–2g of the ESM indicate the sports investigated in each study. Six (6%) studies [35–40] investigated female individuals in the military. Thirty (31%) studies included athletes from multiple different team and/or individual sports. However, seven of these studies did not specify the sports considered. Most (*n* = 48; 75%) studies investigating one sport were individual athlete sports compared to team (*n* = 16; 25%) sports. Endurance runners (*n* = 37; 39%) were mostly included, followed by track and field athletes (*n* = 24; 25%). Noteworthy, however, is that only 3/23 studies [41–43] investigating the MCP with or without HC and MI as contributing factors to injury included endurance runners and track and field athletes. Most of these studies (22/23; 96%) included athletes participating in sports that require multi-directional movements. Conversely, most studies (52/76; 68%) investigating MI with or without MCP and/or HC included endurance running track and field athletes and (18/76; 24%) athletes participating in multi-directional sports. All four studies only related to HC use investigated multi-directional type sports. When HC and MI were investigated, most studies included endurance and track and field athletes.

### Participant Characteristics

The age range for most studies was 13–45 years. Four [44–47] studies included participants aged younger than 13 years and seven [48–54] aged older than 45 years. Half of the studies (*n* = 40) [excluding case studies and case series] did not specify an age range in the inclusion criteria and only reported a mean age or range in the results. None of the studies explicitly stated that peri-menopausal or menopausal female individuals was excluded. None of the studies that included female individuals aged older than 40 years described methods to confirm that the female individuals were not peri-menopausal or menopausal.

Most studies only investigated elite or professional (*n* = 24; 25%) level athletes. Other studies investigated recreational/amateur (*n* = 19; 20%), high school (*n* = 16; 17%), collegiate (*n* = 14; 15%) or a combination of different level (*n* = 15; 16%) athletes. Eight (8%) of the studies did not specify the athletes’ level of participation.

### Injury Definition and Injuries Investigated

No study only considered contact injuries. Most studies (44/96;46%) only included non-contact injuries. Forty-two (44%) did not specify a mechanism of injury in the injury definition, and 10/96 (10%) considered both contact and non-contact injuries. Forty-seven (49%) included time loss as a severity measure, and three (3%) included both time-loss and non-time-loss injuries. Still, almost half (46/96,48%) did not include a time-loss component in the injury definition. According to the International Olympic Committee (IOC) consensus statement, a “time-loss injury” is defined as any injury that results in a player being unable to participate in training or competition for at least 1 day following the day of injury [55]. Seventy studies included multiple injury surveillance methods, while 26/96 (27%) did not report the method of injury data collection or whether the diagnosis was confirmed. Of the 70 studies that specified how injury data were collected or diagnosis confirmed, 52 required diagnosis or confirmation of the injury by medical professionals or medical records, and 45 by imaging. Twenty-seven accepted self-reporting by athletes.

Most studies included upper and lower limb injuries (52/96; 54%). Forty-three (45%) only considered lower limb injuries and only one case study exclusively reported an upper limb injury [56]. Fifty-four (56%) of the studies only included bone or stress fractures. Only one of these studies was not related to MI. Eighteen (19%) of the studies specifically investigated ligament injuries, of which only three included ligaments other than the ACL. Fourteen (15%) studies investigating ligament injuries were related to the MCP. While muscle and tendon injuries might have been included in studies that considered injuries to multiple tissue types, only one study specified the inclusion of muscle or tendon injuries.

### MCP Characteristics

Twenty-five (25/96; 26%) studies investigated MCP with or without MI and HC. Most (18/25; 72%) did not report the definition of eumenorrhea. The remaining studies’ definitions of eumenorrhea varied and were based on one or more parameters, as summarised in Table 1. Similarly, the division of MCP differed, as summarised in Table 2.

**Table 1:** Parameters on which studies based the definition of eumenorrhea

	Number of studies (*n* = 7)	Range
Number of MCPs per year	1	10–12 menses per year[57]
Consistency of MCPs	2	Consistent over 6 consecutive months [58] Occur at intervals of ≥ 38 days [59]
Duration of cycle	2	26–30 days [60] 24–38 days [58]
Duration of menses or other phase	2	Interval from menses to the luteinising hormone surge is consistent to 14 ± 1 days [61] > 8 days of menses [58]

*MCP* menstrual cycle phase

**Table 2:** Differences in the division of the menstrual cycle into sub-phases

MCP	Number and % of studies (*n* = 24)
Follicular/ovulatory/luteal	9 (37.5%) [41, 44, 61–67]
Follicular/luteal/menstruation	4 (17%) [14, 46, 60, 68]
Pre-ovulatory/post-ovulatory	2 (8%) [69, 70]
Other	9 (37.5%) [43, 52, 56, 58, 59, 70–73]

*MCP* menstrual cycle phase

The division of the menstrual cycle in its sub-phases differed between studies and is summarised in Table 2.

Six (6/24; 25%) studies did not report how MCPs were confirmed. Of the remaining studies, 11 [44, 46, 58–63, 66, 68, 74] relied on participant self-reporting. Two of the studies relied on more than one more than one medical test (i.e. blood, saliva or urinary) testing method. Three, two and one studies confirmed MCP by blood, saliva and urinary testing, respectively.

### MI Characteristics

Seventy-four (74/96; 77%) studies investigated MI with or without MCP and/or HC. Several studies included more than one type of MI. Nine (9/74;12%) studies stated MIs were investigated but did not specify the type of MI, while another study [6] pooled amenorrhea, oligomenorrhea and delayed-onset menarche as MI but did not report the definitions of each. Most studies (50/74; 69%) investigated secondary amenorrhea, followed by oligomenorrhea (38/74; 51%), primary amenorrhea (32/74; 43%), luteal-phase deficit (1/74;1%) and one anovulation (1/74;1%). Twenty studies investigated the age of menarche, of which two [47, 75] (10%) considered early-onset menarche and 18 (90%) considered delayed-onset menarche. The definition for early-onset menarche in both studies was a first period younger than 12 years. However, the definition of delayed-onset menarche differed, with studies considering ages ≥ 16 years [76–78], ≥ 15 years [79], and > 14 years respectively as “delayed”.

### HC Characteristics

Fifty-three studies investigated HC use as a contributor to injury risk. Eleven (11/53; 21%) did not specify the type of HC investigated. Three studies included more than one type of HC. Oral contraceptive pills were most commonly investigated (42/53; 79%), followed by injections (4/53; 9%), intrauterine devices (2/53; 37.7%) and one (6%) vaginal ring. Only eight (8/53;19%) of the studies investigating OCPs specified the type or composition of OCP used by athletes. None of the studies considered the different phases of multi-phase contraceptives.

## Discussion

More research is being published on the influence of endogenous and exogenous hormonal fluctuations on female athletes’ injury risk [80]. This scoping review aimed to summarise the characteristics of the literature related to the association between MCP, MI and HC use and MSK injury among female athletes and identify gaps to aid the planning and commissioning of future research.

### Themes

Almost half (48%) of the studies included in this review investigated more than one theme (i.e. MI, CMP and or HC use). Including more than one female hormone-related contributing risk factor acknowledges and reflects the diversity in endogenous and exogenous hormone profiles in female sports teams or training groups. Authors should consider sample sizes that are large enough for a meaningful thematic subgroup analysis. Of the three main themes, the MCP was investigated least (23% of studies). This might be because of the challenges related to MCP research including feasible methods verifying MCPs [27] and athlete barriers to communication about their menstrual cycles. Investigations to identify and address methodological challenges associated with MCP research might result in more MCP-related research in all female athlete domains.

### Study Characteristics

Research about the MCP, HC use and injury in female athletes has increased in the last 5 years. Considering the increase in the popularity of women’s sport (both participation and media coverage), it is fitting that research regarding the MCP and HC use has also increased [80–83]. Whilst the number of articles published in the domain has increased, there is a noticeable lack of research from certain countries across the world. High-income countries such as North America and Europe account for most of the research, with no research from South America and minimal research from Africa, Oceania and Asia. This lack of evidence from low-income to middle-income countries is a common thread among many sports medicine and sports science research areas [84]. Further, only a few articles included data from multiple countries. This lack of multi-national collaboration and multi-site projects is an area for improvement that could aid in pooling research data and potentially increase cohort numbers, thereby accelerating our learning on the topic and reducing research waste [85, 86]. Additionally, consortium-type research will increase the impact of the research and enhance researchers’ understanding of the differences between regions.

Of the included articles, 18% were case studies/case series, which add minimal value to understanding the effect of the MC/HC on MSK injuries, as most of these studies include fewer than ten athletes and simply describe the menstrual function or HC use of these injured athletes. As expected, most studies included cross-sectional/cohort designs, with only one randomised controlled trial. These descriptive studies provide the initial evidence for the associations between MC/HC and MSK injuries. Few studies confirmed MCP by using objective laboratory measures. Most studies used self-reported questionnaires, and it would be useful to understand how accurate the data received from questionnaires are compared to laboratory measures. Future research could investigate interventions to better understand the associations and potential causes [87] relating to MCP and/or HC use and MSK injuries (both all and specific).

More individual than team sports were investigated, and sports such as endurance running, dancing and gymnastics predominated. One explanation for this is that these articles mainly aimed to investigate the FAT or RED-S and did not specifically seek to understand the MCP and HC contribution to MSK injuries. The sports investigated are assumed to have a higher prevalence of RED-S (particularly in female individuals) [88] compared with team sports such as soccer and football. The inclusion of these studies still provides insight into hormonal fluctuation or adjustments in MSK injuries. However, it should be interpreted with caution, as this was not their primary aim.

### Participant Characteristics

Forty studies did not specify an age range in the inclusion criteria and only reported a mean age or range in the results. It is vital to include an age range in research related to ovarian hormone fluctuations as these fluctuations are age dependent. For example, when collecting data using self-reporting questionnaires, women aged older than 45 years might not recognise that they are peri-menopausal and report irregular menstrual cycles that might not be related to training. In cases where studies specified age ranges (< 35 years) or populations, for example high-school or collegiate athletes, in the inclusion criteria, the authors reasonably assumed that the female athletes investigated were not peri-menopausal or menopausal. For clarity, it is however recommended that in future studies that include age ranges that might include early-onset, perimenopausal or menopausal female individuals, authors should specify if and how the athletes’ menopausal status was confirmed.

Most studies only investigated elite or professional (25%) level athletes. A likely explanation is that these athletes operate in more controlled environments, with support staff and resources that can assist with and monitor data collection, making research among this cohort easier. Similar research among adolescent and high school athletes requires more logistical considerations, including obtaining consent from guardians and governing bodies, but is nevertheless essential.

### Injury Definition

Consensus statements on uniform reporting of sports-related injury research have been published in various sports [89–93]. More recently, the IOC consensus statement promotes consistency in defining injury to allow for a comparison of injury-related data in sports [55]. Moore et al. [94], however, highlighted that little of the IOC consensus statement focussed on female athletes specifically and provided further recommendations on methods for recording and reporting epidemiological data on injury in female sports.

In the current literature, large inconsistencies in defining injury exist across studies. Most studies defined injury as “non-contact” injuries (46%), and only 3% of studies reported on time-loss and non-time-loss injuries. These definitions narrow the scope of included injuries and potentially result in underreporting of injury in the current literature. Furthermore, the true association between MCP, MI and HC use on MSK injuries among female athletes could be misrepresented as a result of inconsistent injury definitions used across studies. Studies specifically defining the severity of injuries according to the duration of time lost should use and report a uniform calculation for time lost. For example, is time loss calculated when athletes return to training or competition. The lack of uniform guidelines across sporting codes for collecting and reporting sports injury-related data could explain the variety of injury definitions. Most studies in this review (76%) were published before the guidance provided in the 2020 IOC [55] and 2023 Female Athlete Health Domain [94] consensus statements. Even under uniform reporting guidelines, injury definitions can still be affected by limited resources available to research teams. For example, self-reported injury data should ideally be verified through a clinical assessment or radiological imaging. However, such verifications require more funding and specialised skills and could, therefore, be impractical in most cases. Finally, a lack of awareness of appropriate consensus guidelines regarding the recording and reporting of injury data could result in continued inconsistencies in injury definitions in future studies.

### MCP

The definition of eumenorrhea, the division of MCPs and methods to confirm MCP varied between studies included in the review. The heterogeneity in definitions and methodology limits the possibility of conducting meta-analyses, resulting in confusion and possible misinterpretation of results [26, 27]. Two recent publications [26, 27] proposed standardised guidelines and methods for studying the MCP as an independent variable in an attempt to catalyse the accumulation of knowledge on the physiological and psychological effects of the menstrual cycle. Most of the MCP studies in this review were published before these guidelines were published. As might be the case with applying standard injury definitions, standard MCP definitions and methods might be affected by financial resources, technology and skills available to research teams. Confirming MCP using blood tests for example might be the gold standard but impractical in most settings. The importance of evaluating the ecological validity of methods used to verify the MCP should be considered in future research endeavours. Researchers should rely on accurate measurement rather than estimation to reach valid and significant conclusions in a topic that is characterised by substantial controversy and conflicting findings [95]. We suggest, in agreement with a recent editorial’s authors [95], that if measurements were not taken, authors should provide a justification for this decision, alongside a transparent explanation of the limitations in their study design, and a comprehensive disclosure of the effects of their assumptions and estimates on the research quality, and the ability to make scientific inferences, and the specific clinical risks associated with these assumptions and estimates. In situations where financial and administrative constraints hinder the availability of ample resources, it may be prudent to opt for a classification system that simply relies on the distinction between bleeding and non-bleeding phases, as this may be the most straightforward and accurate approach. Research investigating the most practical yet accurate methods to explore the influence of MCP on MSK health or reasons for not implementing currently published guidelines might yield valuable insights for future research designs.

While the role of MCPs in injury risk is gaining more recognition, the distribution of research across different types of injuries and sports is unbalanced. For example, the association between MCPs and ACL injury and laxity has been investigated extensively [1, 8, 9]. However, ankle ligament injuries are more or equally prevalent compared to knee injuries among female netball [96, 97], basketball [98] and football [99] players. Additionally, instability-type injuries of the shoulder [100] and ankle [15] are higher among female than male athletes competing in gender-comparable sports. Still, studies investigating the association between ankle and shoulder injuries and MCPs are scarce compared to ACL-related studies.

### MI

Like MCPs, the definition of MI could have been more consistent among studies. Some studies, for example, pooled amenorrhoea, oligomenorrhea and delayed-onset menarche as “MI”, while others analysed and reported them as individual MIs. The definition of delayed-onset menarche also varied. A possible explanation might be that “normal” ages of menarche differ between countries, races and ethnicities [101].

Most of the research investigated the association between MI (as an individual risk factor or as part of FAT/RED-S) and bone injuries. Evidence related to the association between MI and soft-tissue injuries remains scarce. Additionally, MI is characterised by oligomenorrhoea, polymenorrhoea, amenorrhoea, anovulatory or luteal-phase deficient cycles [6, 8, 17]. While some studies include all of these conditions in the definition of MI, others only include, for example, amenorrhea and oligomenorrhea. This could be due to the practicality and costs of methods required to confirm luteal-phase deficiency and anovulation. A feasibility study to establish if luteal-phase deficiency or anovulation affects injury risk before conducting full-scale studies might be valuable.

### HC Use

The potential impact of HC on the MSK system in athletes is an area of ongoing research with varied findings [22, 102]. Most of the studies included in this review (79%) investigated the effect of OCP on injury risk but the influence of other forms of HCs, including patches, intrauterine devices and vaginal rings, are less prevalent. As mentioned, most research in this review has been conducted in North America and Europe. Hormonal contraceptive prescription practices and subsequent athlete use in other countries might be different. Therefore, future research related to athletes’ HC use characteristics in other countries and the effect of HC other than OCPs on MSK injury risk is warranted.

Some studies (21%) did not specify the type of HC investigated while others investigated more than one type of HC, but pooled them for a statistical analysis. Hormonal contraceptives have different exogenous hormone compositions, are processed via different physiological systems, and can have local or systemic effects [103]. Their effect on athletes’ bone [104] and soft-tissue structures and function [21, 105] and, in turn, injury risk could be different. Therefore, it is important to specify the type of HC investigated when reporting on the effect of HC on injury risk.

In this review, HC research mainly focussed on bone/stress fractures. Research investigating the effect of OCP on soft-tissue injuries is scarce. While HC may theoretically influence bone density [104], connective tissue health [21], and muscle physiology and function [102, 105], the direct impact on MSK injury risk remains uncertain. Factors such as age, individual hormonal profiles, type of contraceptive and lifestyle factors (including exercise habits) can all contribute to variations in how HCs may affect MSK health [21, 106, 107]. Further research is needed to understand these complex interactions better (Table 3).

**Table 3:** Summary of gaps identified and future research recommendations related to the influence of MCP, MI and HC use on injury

Research characteristics	Gaps identified/future research recommendations
Geography	Research in Oceania, Africa, South America and Asia Research to identify barriers to MCP, MI and HC-use research in Oceania, Africa, South America and Asia Multi-site research across several countries
Type of sport	Team sport athletes
Participants	Research among non-professional/elite athletes especially adolescent high-school athletes
Injury definition	Research to identify barriers to implementing consensus statement definitions of injury in research
MCP	Investigations to identify and address methodological challenges related to MCP research might result in more MCP-related research in all female athlete domains Validity of self-reporting of MCP, i.e. establishing the most accurate ecologically valid methods of classifying and confirming MCPs If measurements were not taken, authors should provide: (a) a justification for this decision (b) a transparent explanation of the limitations in their study design (c) a comprehensive disclosure of the effects of their assumptions and estimates on the research quality Associations between MCP and effects on muscle, tendon and ligament (other than the ACL) injuries
MI	Guidelines on defining and reporting MI Evidence related to the association between MI and soft-tissue injuries
HC	Research on the effect of HC methods, other than OCP, on injury risk among female athletes Research to better understand the complex interactions between HC phase, individual hormone profiles and exercise habits
Summative research	Future systematic reviews with narrower inclusion criteria that assess risk of bias and study quality can significantly enhance researchers’ and practitioners’ understanding of the topic by providing more precise and reliable insights

*ACL* anterior cruciate ligament, *HC* hormonal contraceptive, *MCP* menstrual cycle phase, *MI* menstrual irregularities, *OCP* oral contraceptive pill

## Strength and Limitations

This scoping review is not without limitations. Scoping reviews commonly do not include a quality appraisal of the individual studies and this was not the main purpose of this review. Knowing the quality of individual studies is important when assessing and comparing the rigour of specific studies or groups of studies. The findings of this scoping review will inform future systematic reviews with narrower inclusion criteria that assess the risk of bias and study quality can significantly enhance researchers’ and practitioners’ understanding of the topic by providing more precise and reliable insights.

It is possible that we may have missed available data because of excluding studies published in languages other than English, which, in turn, might have impacted the geographical location of the results presented. While we can comment on the lack of studies that use recommended surveillance strategies, in alignment with scoping review methodology we did not evaluate the risk of bias or assess the methodological quality of the included studies, and thus cannot directly comment on low-quality research.

## Conclusions

Research related to MCP, MI and HC as contributing MSK injury risk factors is increasing. However, several gaps have been identified, including research from countries other than North America and Europe, among non-professional/elite-level athletes, in team sports and specific injuries related to MCP, MI and HC, respectively. Differences in the methodology and terminology of injury, MCP and MI hinder comparative and summative research, and future research should consider current published guidelines during the study design. Identifying barriers to following standard guidelines or research investigating the most practical yet accurate methods to investigating the influence of MCP on MSK health might yield valuable insights for future research designs. Collaborative international multi-site projects could be used to pool research talent, enterprise, and data and potentially increase cohort numbers, thereby accelerating our learning on the topic.

## Supplementary Information

Below is the link to the electronic supplementary material.

Supplementary file1 (DOCX 356 KB)

## Declarations

### Availability of data and material

The data and materials that support the findings of this study are included within the article. No additional data or materials are available.
