# Supplementary material for: The Association Between Menstrual Cycle Phase, Menstrual Irregularities, Contraceptive Use and Musculoskeletal Injury Among Female Athletes: A Scoping Review
Source: Sports Med. 2024 Aug 31;54(10):2515–30. doi: 10.1007/s40279-024-02074-5 (PMC11467081; doi:10.1007/s40279-024-02074-5)
Supplement: Supplementary file 1 — Supplementary file1 (DOCX 356 KB) [file 40279_2024_2074_MOESM1_ESM.docx]

**JOURNAL: SPORTS MEDICINE**

**TITLE: THE ASSOCIATION BETWEEN MENSTRUAL CYCLE PHASE, MENSTRUAL IRREGULARITIES, CONTRACEPTIVE USE AND MUSCULOSKELETAL INJURY AMONG FEMALE ATHLETES: A SCOPING REVIEW**

**RUNNING TITLE: MENSTRUAL CYCLE PHASE, IRREGULARITIES, CONTRACEPTIVE USE AND MUSCULOSKELETAL INJURY IN ATHLETES**

**AUTHORS**

Candice MacMillan^1,2^ Benita Olivier^3,4^ Carel Viljoen^5^ Dina C Janse van Rensburg^6^ Nicola Sewry^2,7^

1. Department of Physiology, Faculty of Health Sciences, University of Pretoria, Pretoria, South Africa.
2. Sport, Exercise Medicine, and Lifestyle Institute (SEMLI), Faculty of Health Sciences, University of Pretoria, Pretoria, South Africa
3. Centre for Healthy Living Research, Oxford Institute of Allied Health Research, Department of Sport, Health Sciences and Social Work, Oxford Brookes University, Oxford, United Kingdom
4. Wits Cricket Research Hub for Science, Medicine and Rehabilitation, Department of Physiotherapy, School of Therapeutic Sciences, Faculty of Health Sciences, University of the Witwatersrand, Johannesburg, South Africa
5. Department of Physiotherapy, Faculty of Health Sciences, University of Pretoria, Pretoria, South Africa.
6. Section Sports Medicine, Faculty of Health Sciences, University of Pretoria, Pretoria, South Africa.
7. International Olympic Committee (IOC) Research Centre, Pretoria, South Africa.

**Supplementary material Figure 1:**


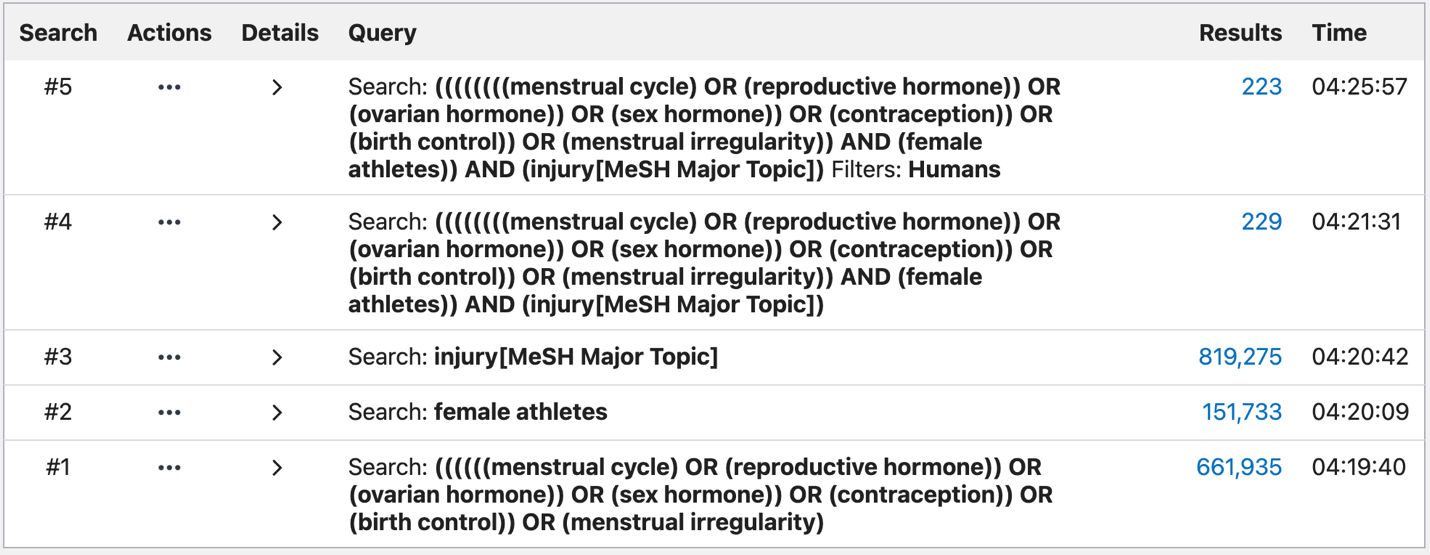


**Supplementary Figure 1:** PubMed Search strategy using keywords related to “menstrual cycle”, “contraception”, “injury”

**Supplementary Table 1:** Overview of eligibility criteria

| Population | Included:   - Human female *athletes* (defined as one who (i) actively engages in individual or team sports training, where the main motivation or goal is to improve sport-specific skills, performance, or results (technical, physical or tactical) for competition (ii) regularly competes against others unless injured or in competition break; (iii) formally registered in a local, regional or national sport federation (iv) have sport training and competition as his/her main physical activity or focus of personal interest, devoting several hours per week or more, depending on the phase of season/competition)[1]. - Active female recruits in the military. - Female *exercisers* (defined as one who engages in physical activity with the will to: (i) augment their fitness level; (ii) improve their health; (iii) ameliorate their physique, and (iv) acquire or improve skills)[2] of reproductive age (ie., postmenarche and premenopausal) will be included. - Eumenorrheic women, women with menstrual irregularities (e.g., oligomenorrhoea, polymenorrhoea, amenorrhoea, anovulatory and luteal phase deficient cycles), and HC users (e.g., combined and progestogen-only OCPs, injections, implants, patches, intra-uterine systems) were included.   Excluded:   - Pregnant, perimenopausal and menopausal females were excluded. - Participants who have used any medication known to affect ovarian hormone profiles (except for HCs) or the musculoskeletal system. |
| --- | --- |
| Concept | Studies investigating both exposures affecting the endogenous reproductive hormone status of the participants (i.e. MC phases, associated disturbances, and HCs) and sporting injuries were included. An injury was defined as musculoskeletal tissue damage or other derangement of normal physical function due to participation in sports, resulting from rapid or repetitive transfer of kinetic energy.[3] |
| Context | Females participating in any individual or team sport, at any level (elite, amateur, recreational, high school, collegiate), in any country were included. |
| Study designs | This scoping review considered both experimental and quasi-experimental study designs, including randomised controlled trials, non-randomised controlled trials, before and after studies and interrupted time-series studies. In addition, analytical observational studies including prospective and retrospective cohort studies, case-control studies and analytical cross-sectional studies was considered for inclusion. Descriptive observational study designs including case series, individual case reports and descriptive cross-sectional studies for inclusion.  Observational studies will be considered for inclusion if they meet the following inclusion criteria: (i) published, in full, in a peer-reviewed journal; (ii) have the objective of assessing injury risk in response to MCP, MI and/or HC use and (iii) report the incidence of injuries aligned with phases of the MC and/or menstrual regularity and/or HC usage.  Experimental studies will be considered for inclusion if they are: (i) published, in full, in a peer-reviewed journal (ii) have the objective of assessing the efficacy of measures employed to mitigate MC and/or HC related injury risk (iii) report the change in injury incidence after implementation of the specific preventative strategies.  Qualitative studies were not included in this review. |

MI = Menstrual irregularity; HCU = Hormonal contraceptive use; MC= menstrual cycle; MCP = Menstrual cycle phase

Supplementary Table 2a: Studies investigating the association between menstrual cycle phases (MCP) and musculoskeletal injury.

| **Author and Year** | **Study Aim** | **Sport/Activity** | **Summary of main findings** |
| --- | --- | --- | --- |
| Adachi et al. 2008[4] | Determine association between ACL injuries and MCP in teenaged female athletes. | Multiple including Basketball; volleyball, handball | Significant increase in non-contact ACL injuries in teenage female athletes during the ovulatory phase of the menstrual cycle. |
| Bambaeichi et al. 2010[5] | Investigate rate of female athletes’ injuries in different MCP. | Multiple unspecified | The rate of injuries, ligament injury and ACL injuries were significantly higher in the luteal phase, compared to ovulation and follicular phases. When data were analysed based on the duration of each phase, more injuries were observed in the ovulation phase. |
| Beynnon et al. 2006[6] | Establish if the likelihood of suffering an ACL injury is affected by the MCP. | Alpine skiing | ACL injury significantly more likely in preovulatory compared to post-ovulatory phase. Menstrual history data found similar results, but the difference was not statistically significant. |
| Lago-Fuentes et al. 2021[7] | To describe and compare the injury incidence to MCP in professional futsal players over two consecutive season and, secondly, compare injury risk between MCPs. | Futsal | This study suggests the relevance to track the MC, but reduces its possible relationship or influence on the injury distribution during each phase of the MC. |
| Lefevre et al. 2013[8] | Describe the distribution of ACL lesions according to MCP in a large population of female recreational skiers. | Alpine skiing | ACL tear was 2.4-fold more likely in pre- (follicular and ovulatory) than post-ovulatory phase. ACL tears were more frequent in pre-ovulatory phase, whether in patients using OCP or another contraceptive methods. |
| Martin et al. 2021[9] | Investigate the association between injury incidence and MCP and length of menstrual cycle. | Football/Soccer | Injury incidence rates were 47 and 32% greater in the late follicular phase compared to the follicular phase and luteal phase, with muscle and tendon injury incidence rates in the late follicular phase being almost double that of the other phases. Furthermore, a relatively large proportion of all injuries (20%) occurred after the expected date of menstruation. |
| Pelkowski et al. 2020[10] | Description of monozygotic twin sisters sustained ACL injuries within 48 hours of one another. | Basketball | Both were in ovulatory phase, and had a regular menstrual cycle. |
| Reyes et al. 2023[11] | Describe the relationship between the MCPs and the ACL injury. | Football/Soccer | Most injuries occurred during the luteal phase and menstruation. |
| Sommerfield et al. 2020[12] | Examine sports and physical education injury rates in young females during a school year and to investigate if an association exists between injury and phase of the menstrual cycle. | Multiple unspecified | There was no significant association between the stage of the menstrual cycle and the likelihood of injury (P = 0.18). |
| Stijak et al. 2014[13] | Determine effect of concentrations of testosterone, 17-b estradiol and progesterone between female patients with and without ACL rupture. | Track and field; Football/Soccer; Other: handball, volleyball | Young female athletes with lower concentrations of sex hormones are more prone to ACL rupture which is why they need to reduce their sports activities during the pre-ovulatory phase of the menstrual cycle, when these concentrations are additionally reduced. |

MCP = Menstrual cycle phase; ACL = Anterior cruciate ligament; OCP = Oral contraceptive pill

Supplementary Table 2b: Studies investigating the association between menstrual irregularities (MI) and musculoskeletal injury.

| **Author and Year** | **Study Aim** | **Sport/Activity** | **Summary of main findings** |
| --- | --- | --- | --- |
| Ackerman et al. 2014[14] | Compare fracture prevalence in oligo-amenorrheic,  eumenorrheic, and non-athletes and determine relationships with bone density, structure, and strength estimates. | Athletes ran at least 20 miles every week or were engaged in weight-bearing aerobic activity or at least 4 hours/week for at least 6 months. | Weight-bearing athletic activity increases bone mass density, but may increase stress fracture risk in those with menstrual dysfunction. |
| Armstrong et al. 2004[15] | Compare men and women at the US Naval Academy who sustained lower extremity stress fractures during a military summer training program with a matched group of uninjured recruits to identify factors that may increase the risk factors (including female athlete triad). | Military | Among female plebes, there was no evidence of the female athlete triad (eating disorders, menstrual dysfunction, or low bone density) and is therefore not a risk factor for stress fracture. |
| Barrack et al. 2014[16] | Identify risk factors, including MC dysfunction, associated with stress reactions and stress fractures, among exercising girls and women. | 2 h/week of purposeful exercise as documented in a 4-week exercise log at baseline | Risk of bone stress injury increased from 15% to 20% for single risk factors and to 30% to 50% for combined female athlete triad–related risk factor variables. |
| Beals et al. 2002[17] | Determine if there is an increased incidence of menstrual dysfunction and MSK injuries among female athletes identified with disordered eating. | Track and field; Endurance running; Gymnastics; Cheerleading; Basketball; Football/Soccer; Diving, golf, volleyball, swimming, waterpolo, tennis, softball, field hockey. | Although the difference failed to reach significance athletes reporting MI suffered more bone and muscle injuries (40.0% and 67.4%, respectively) during their collegiate career compared to those with normal menses (31.4% and 60.8%, respectively). |
| Bennell et al.1995[18] | To investigate risk factors (including MI) for stress fractures in Track and Field athletes. | Track and field | Women with a history of stress fracture were significantly more likely to have a history of oligomenorrhea. No significant difference between the two groups in the frequency of amenorrhea, duration of amenorrhea for >6months. Menarche after 14 years of age or dietary restriction to maintain weight. Those with oligomenorrhea had 6x greater risk of sustaining a stress fracture than those without oligomenorrhea. |
| Benson et al.1989[19] | Investigate the relationship between nutrient intake, body mass index, menstrual function, and ballet injury. | Dancing; Ballet – minimum of 10 class hours of dance per week | Dancers with abnormal menses had more bone injuries than normally menstruating dancers. |
| Cooper et al. 2005[20] | Present a case of a female Cross-country Runner with femoral neck stress fracture | Endurance running | This case demonstrates the importance of screening for amenorrhea and disordered eating when seeing a female patient with a stress fracture and the importance of not only asking about current menstrual history and weight, but to ask about previous menstrual history and weight. |
| Constantinou et al. 2021[21] | Present a case of bilateral navicular stress fractures in a juvenile patient. | Track and field | With regard to history, she reported having an irregular menstrual cycle. |
| Edama et al. 2021[22] | Clarify the relationship between the triad risk assessment score and the sports injury rate in 116 female college athletes. | Track and field; Endurance running; Basketball; Football/Soccer; Swimming, Volleyball | Significantly more athletes at moderate and high-risk categories (that include amenorrhea and delayed menarche) and had bone stress fractures and bursitis than athletes at low risk. |
| Fryar et al. 2023[23] | Evaluate the impact of two of the female athlete triad components, MI and disordered eating leading to low energy availability, on (1) the number and location of time loss injuries and (2) injuries that required surgery. | Gymnastics | Those who reported disordered eating had higher injury rates than those with MI. The female athlete triad can increase overall injury risk and can also have lifelong impacts beyond MSK injuries. |
| Goolsby et al. 2012[24] | Demonstrate the potential serious consequences of the female athlete triad and its effects on bone. | Endurance running | This injury could have been prevented if the signs and symptoms of her injury had been addressed and there had been better knowledge of her risk factors. |
| Haddad et al. 1997[25] | Present a case of a displaced stress fracture of the femoral neck in an active amenorrhoeic adolescent. | Endurance running | Although patients' endocrine work up was unremarkable should be noted clinical history includes primary amenorrhea. |
| Heikura et al. 2018[26] | Report energy availability (EA), metabolic/reproductive function, bone mineral density, and injury/illness rates in national/world-class female and male distance athletes. | Track and field; Endurance running; Race walkers | Bone injuries were 4.5-fold more prevalent in amenorrhoeic. |
| Hutson et al. 2021[27] | Investigate the association between menstrual function and BSI (Bone stress injuries) and explore whether plyometric training may protect against BSI in individuals with menstrual disturbances. | Endurance running | BSI incidence rate was similar in oligo/ amenorrhoeic and eumenorrhoeic runners that did plyometric training, but 3.78 (p = 0.001, 95% CI: 1.68–8.5) times greater in oligo/amenorrhoeic vs eumenorrhoeic runners that did not. OA vs EUM 2.25x higher for BSIs without accounting for plyometric training. |
| Ihalainen et al. 2021[28] | Determine body composition, energy availability, training load, and menstrual status in young elite endurance running athletes and, in a secondary analysis, to investigate how these factors differ between non-running controls, and amenorrhoeic and eumenorrheic athletes. | Endurance running | More than half of this group of runners was amenorrhoeic, and they were injured more and ran less than their eumenorrheic counterparts. And AME had more injury days than EUM. |
| Johnson et al. 2001[29] | Describe an unusual cause of persistent low back/sacroiliac pain: a fatigue-type sacral stress fracture. | Endurance running; Basketball; Football/Soccer; Other: Eight athletes engaged in different sports: 1x soccer, 1 basketball, 6 endurance runners | The most significant risk factor for fatigue-type sacral stress fractures was an increase in impact activity due to a more vigorous exercise program. Potential risk factors such as abnormal menstrual history, dietary deficiencies, and low bone mineral density were examined. The clinical course was protracted, with an average 6.6 months of prolonged low back pain before resolution of symptoms. Sacral fatigue-type stress fractures did not preclude the athletes from returning to their previous level of participation once healing had occurred. |
| Kandemir et al. 2018[30] | Evaluate and compare the effects of anorexia nervosa and exercise-induced amenorrhea on bone parameters and to identify groups at high risk for fracture. | Endurance running; Other: weight-bearing exercise | A high stress fracture rate in normal-weight oligomenorrheic athletes, who have comparable bone mineral density measures to controls, indicates that bone mineral density in these women may need to be even higher to avoid fractures. A higher proportion of OAs than AN and controls had stress fractures. |
| Korpelainen et al. 2001[31] | Identify factors predisposing athletes to multiple stress fractures with the emphasis on biomedical factors. | Endurance running; Other: Long distance running, sprinting, jumping, orienteering, cross-country skiing, power events, ball games | Nearly half (40%) of the females with stress fractures reported menstrual irregularities. |
| Laframboise et al. 2013[32] | Illustrate the varying presentations of the female athlete triad (FAT) in four athletes who sustained fractures or stress fractures. | Track and field; Endurance running; Dancing | The case series concluded that the female triad may be a tetrad of coexisting conditions including disordered eating, menstrual dysfunction, low bone mineral density, and endothelial dysfunction. OCP medication may be considered to prevent further loss of bone mineral density in athletes with functional hypothalamic amenorrhea over the age of 16 and only if bone mineral density is decreasing despite nutrition. |
| Lambert et al. 2020[33] | Characterise bone health in relation to stress fracture history, body composition, eating disorder risk, and blood biomarkers in professional male and female ballet dancers. | Dancing | There was no difference in bone mass density or frequency of stress fracture between dancers who had irregular cycles and those who did not. None of the female dancers in the present investigation reported amenorrhea. |
| Myer et al. 2009[34] | To present a unique case of a young pubertal female athlete who was prospectively monitored for previously identified ACL injury risk factors for three years before sustaining an ACL injury. | Endurance cycling; Basketball | The athlete reported that menarche occurred at age 12, between the measurements for years 2 and 3. At the interview, she indicated that she had missed 3 successive menstrual cycles at the time of injury, with more than three months since the last menses. |
| Native et al. 2013 | To examine the relationships between MRI grading of bone stress injury with clinical risk factors (including MI and HC use) and time to return to sport in collegiate track and field athletes | Track and field; Endurance running | Among the female athletes, MRI grade was significantly higher among those classified with oligo/amenorrhea compared to eumenorrhea. Fracture history, menstrual status, age at menarche, and gender were not significantly associated with time to return to sport. |
| Nose-Ogura et al. 2018[35] | To examine whether the Triad increases the risk of stress fractures, athletes were assigned to a “teenage” group and a “20s” group. | Multiple unspecified | The percentage of stress fractures in the amenorrhoeic group (22/120, 18.3%) was significantly higher than that in the EA group (14/180, 7.8%, p<0.0001). increased odds of SF. |
| Okamoto et al. 2010[36] | Case of a displaced stress fracture of the femoral neck in an adolescent female distance runner with amenorrhea. | Endurance running | Amenorrhea might have been a contributing factor to athletes’ stress fracture. |
| O'Leary et al. 2023[37] | Investigate associations between menstrual function, eating disorders, and risk of low energy availability with MSK injuries in British Service women. | Military | Menstrual disturbances (oligomenorrhoea/amenorrhoea, history of amenorrhoea, and delayed menarche) were not associated with injury. |
| Prather et al. 2016[38] | To determine the prevalence of stress fractures within the previous 2 years, menstrual dysfunction one year following the onset of menses, and disordered attitudes towards body perception and eating attitudes in an elite group of female soccer athletes. | Football/Soccer | There was no difference in the reported incidence of stress fractures between athletes with menstrual dysfunction as compared to those with no menstrual dysfunction. |
| Steinberg et al. 2013[39] | Investigate the association between joint range of motion, anatomical anomalies, body structure, dance discipline, and injuries in a large sample of female recreational dancers and to identify potential risk factors (including age of menarche) for injury. | Dancing | An association was found between early age of onset of menarche and rate of injuries 38% of the dancers who had an early age of onset of menarche were injured versus 45% of the dancers with an average (12–14 years) or late (>14 years) age of onset of menarche. |
| Tenforde et al. 2011[40] | Evaluate lifetime prevalence and risk factors for overuse injuries in high school athletes currently participating in long-distance running. | Track and field; Other: cross country running | No differences in menstrual history, including age of menarche or menstrual irregularities, were seen in female subjects with a history of injury. |
| Thein-Nissenbaum et al. 2011[41] | Determine the association between disordered eating,  menstrual dysfunction (MD), and MSK injury (in females participating in various high school-sponsored sports. | Track and field; Gymnastics; Cheerleading; Dancing; Basketball; Football/Soccer; Other: diving, cross country, tennis, volleyball, swim, softball, golf | There was no relationship between MD and MSK injury. |
| Thein-Nissenbaum et al. 2012[42] | To determine the prevalence of and relationship between MI and injury in high school athletes. | Track and field; Endurance running; Endurance cycling; Gymnastics; Cheerleading; Dancing; Football/Soccer; Other: diving, cross country, tennis, volleyball, swimming, golf | Athletes with MI reported a greater percentage of severe MSK injuries than did athletes with normal menses. |
| Tomten et al. 1996[43] | To estimate the prevalence of athletic amenorrhea in a wide range of long-distance runners and the relation to possible risk factors. | Endurance running | The runners with menstrual disorders had a significantly higher incidence of stress fractures. |
| Vajapey et al. 2019[44] | Reporting on sacral stress fractures among. | Endurance running; Other: cross country runner | 1 of the 3 female runners who in the case-series had irregular menses. |
| Voss et al.1997[45] | To report on an unusual case of bilateral femoral stress fractures in an amenorrhoeic female athlete. | Triathlon | Hormonal disorders may increase the risk for fatigue fractures. |
| Wilson et al.1994[46] | To report on the case of a 30-yr-old amenorrhoeic athlete with significantly reduced bone density who developed stress fractures and then sustained a full fracture of the humerus. | Endurance running | Bone loss that occurs in amenorrhoeic athletes may be severe enough to lead to osteoporotic fractures many years before the menopause and therefore this condition should not be regarded as benign. |
| Witkos et al. 2022[47] | Determined whether menstrual cycle disorders occurring in female swimmers overlap with the occurrence of sports injuries. | Swimming | Disturbances in the normal menstruation cycle, occurring among a certain percentage of the swimmers, positively correlated with the number of injuries that were recorded among these athletes. |
| Wolman et al. 1989[48] | Report on a 17 year old runner with primary amenorrhoea who had a slipped upper femoral epiphysis. | Track and field | Female athletes with primary amenorrhoea and pubertal delay are probably at high risk of these disorders. |

BSI = Bone stress injuries; MI = Menstrual irregularity; ACL = Anterior Cruciate ligament; MD = Menstrual dysfunction; MSK = Musculoskeletal; FAT = Female athlete triad; AME = amennorhoea; EUM = eumennorhea; OA = oligomenorrheic athletes; AN =anorexia nervosa; OCP = Oral contraceptive pill; HC = hormonal contraceptive; EA = eumenorrehea athlet

| Supplementary Table 2c: Studies investigating the association between hormonal contraceptive use and musculoskeletal injury. | | | |
| --- | --- | --- | --- |
| **Author and Year** | **Study Aim** | **Sport/Activity** | **Summary of main findings** |
| Agel et al. 2006[49] | To determine if the use of OCPs affects the rate of non-contact ACL injury and ankle sprains. | Basketball; Football/Soccer | No difference in the rate of injuries between athletes using hormonal therapy and not using hormonal therapy. |
| Liederbach et al. 2008[50] | Compare selected intrinsic and extrinsic variables (including HC use) between the dancers who experienced ACL injury with those who did not. | Dancing | No difference between injured and non-injured women with regard to OCP use. |
| Niyonsenga et al. 2013[51] | Establish risk factors (including OC use) for soccer injuries among first-division Rwandan female soccer players. | Football/Soccer | OC use was associated with injury. |
| Redinger et al. 2023[52] | To identify if competitive heavyweight female rowers who were OC users had altered bone quality compared with their teammates who did not use OCs. | Rowing | The self-reported stress fracture incidence between OC users and non-OC users was 21.4 and 22.9%, respectively. Additionally, both OC groups and fracture groups were similar in respect to their self-reported age at menarche. |

OCP = oral contraceptive pill; OC = oral contraception; ACL=Anterior cruciate ligament; HC = Hormonal contraception

| Supplementary Table 2d: Studies investigating the association between menstrual cycle phase and hormonal contraceptive use and musculoskeletal injury | | | |
| --- | --- | --- | --- |
| **Author and Year** | **Study Aim** | **Sport/Activity** | **Summary of main findings** |
| Arendt et al. 2002[53] | Analyse 28-day periodicity of non-contact ACL injuries in athletes using and not using OC. | Basketball; Multiple unspecified | Significant relationship between the occurrence of non-contact ACL injuries and MC (earlier in cycle) regardless of OC use status. |
| Dragoo et al. 2011[54] | To investigate whether collegiate female athletes (HC users and athletes who do not use HC) with elevated serum relaxin concentrations (SRC) sustain ACL tears at an increased rate compared with those with lower SRC. Fluctuations in SRC is related to MCP. | Gymnastics; Basketball; Football/Soccer; Lacrosse, field hockey, volleyball | Elite female athletes (HC users and athletes who do not use HC) with ACL tears have higher SRC (during late luteal phase) than those without tears. Those with an SRC greater than 6.0 pg/mL had over 4 times increased risk for a tear. HC use was not associated with ACL tears, and controlling for HC use led to larger differences with SRC levels and ACL tears. |
| Myklebust et al. 1998[55] | Injury mechanisms of ACL injuries and possible risk factors including menstrual status and hormonal contraceptive use. | Handball | The researchers also spilt the HC users time of injury into MCP’s and not HC-phase and concluded that most injuries occurred in the late luteal phase. There may be an increased risk of ACL injury during the week prior to or after the start of the menstrual period. |
| Moller-Nielsen et al. 1989[56] | To investigate women soccer players traumatic injuries in premenstrual and menstrual phases during the rest of the cycle. Also regarding contraceptive use. | Football/Soccer | Increase in injury rates in premenstrual/menstrual vs rest. Still held true when separated out into HC use and not. |
| Ruedl et al. 2009[57] | The objective of this study is twofold: (1) to investigate a possible protective effect of OCP use against ACL injuries in recreational skiers and (2) to compare the frequencies of non-contact ACL injuries in the preovulatory phase with that in the postovulatory phase of the menstrual cycle in recreational skiers. | Alpine skiing | Of the ACL-injured skiers, 57% were in the preovulatory phase at the time of injury, whereas 41% of the controls were in the preovulatory phase at the time of questioning. An odds ratio of 1.92 (CI: 1.07–3.44) was calculated. Regarding subjects and controls without OC use, only a trend (P = .084) toward a 1.88 (CI: 0.92–3.88) increase in ACL injuries in the preovulatory phase was detected. |
| Slauterbeck et al. 2002[58] | To determine if ACL injuries in female athletes occur randomly or correlate with a specific MCP. | Multiple unspecified | ACL injuries occurred most frequently on days 1 and 2 of menses, suggesting that ACL injury is not random but occurs more often around the time of menses. 16% of athletes injured their ACLs while on OCPs. Researchers included OCP users in the main sample statistical analysis i.e. also divided time of injury according to MCP and not HC. |
| Wojtys et al. 1998[59] | To investigate the variation in ACL injury rates during the female monthly cycle. | Basketball; Football/Soccer; Karate, skiing, softball, volleyball, tennis | A significant association was found between the stage of the menstrual cycle and the likelihood for an ACL injury (P=0.03). There were more injuries during the ovulatory phase of women with regular cycles (days 10 to 14) than expected. In contrast, significantly fewer injuries occurred during the follicular phase (days 1 to 9) (4 injuries; 13% observed, 32% expected). Because of the small number of subjects who used OCPs (N=5), they were unable to stratify by contraceptive use in the analysis. |
| Wojtys et al. 2002[60] | To determine the association between MCP and ACL tears. | Multiple unspecified | Increased susceptibility for ACL injury in the ovulatory phase of the menstrual cycle in women who did not use OCPs. OCP use appears to diminish the disproportionate distribution of ACL injuries during the menstrual cycle |

OC = oral contraception; ACL=Anterior cruciate ligament; HC = Hormonal contraception; MCP = Menstrual cycle phase; SRC = serum relaxin concentrations

| Table 2e: Studies investigating the association between menstrual irregularity and hormonal contraceptive use and musculoskeletal injury | | | |
| --- | --- | --- | --- |
| **Author and Year** | **Study Aim** | **Sport/Activity** | **Summary of main findings** |
| Barrow et al. 1998[61] | Determine the prevalence of stress fractures and its relationship to menstrual history in a sample of collegiate female distance runners. | Track and field; Endurance running | Females distance runners who have a history of irregular or absent menses and who never used OCs may be at increased risk of developing a stress fracture. |
| Bennell et al.1996[62] | To investigate risk factors (including MI) for stress fractures in Track and Field athletes. | Track and field | Female athletes who sustained stress fractures had a significantly later age of menarche, fewer menses in the year preceding the study, and lower menstrual index, indicating fewer menses per year since menarche.  No difference between female groups for years since menarche, participation in premenarcheal training, and past or current use of OCP. |
| Cheng et al. 2021[63] | Establish the relationship between MI, HC-use and bone stress injuries among collegiate athletes in the US. | Track and field; Endurance running; Gymnastics; swimming, diving | The prevalence of primary amenorrhea was higher in athletes with stress fractures than in those without stress fractures. OCP use was associated with lower odds of having a history of stress fractures. Compared with OCP use, injectable HC use was significantly associated history of stress fractures. No association between past MI and a history of stress fractures. |
| Cline et al.1998[64] | To identify characteristics and factors associated with increased risk for stress fractures in military women. | Military | No relationship was evident between bone density values and stress fractures in amenorrheic soldiers. 63% of injured subjects were on OCP prior to entering the Army. |
| Duckham et al. 2012[65] | To identify psychological and physiological correlates of stress fracture in female endurance athletes. | Endurance running; Triathlon | The prevalence of current a/oligomenorrhoea and past amenorrhoea was higher in athletes who sustained stress fractures than in control groups. This remained even when adjusted with HC use. |
| Duckham et al. 2015[65] | The purpose of this study was to determine the annual incidence of SF in female endurance athletes based in the United Kingdom. | Endurance running; Triathlon | In two stress fracture cases women were eumenorrheic with no history of menstrual dysfunction or HC use. 1 of the two had late onset menarche (16 years). |
| Dugowson et al. 1991[66] | Present a case of a non-traumatic femur fracture in an amenorrhoeic athlete. | Endurance running | The study demonstrates the contributing effect of amenorrhea on the outcome (i.e. femur fracture) following minor trauma. |
| Eller et al. 1997[67] | Present a case study of amenorrhoeic female athlete with a sacral stress fracture. | Endurance running | The female athletes with sacral stress fractures were amenorrheic and were taking OCPs for the treatment of amenorrhea. |
| Gehman et al. 2022[68] | To explore whether energy availability, menstrual function, measures of bone health, and a modified Female Athlete Triad Cumulative Risk Assessment (CRA) tool are associated with a history of multiple BSIs. | Endurance running; | There were no differences in prevalence of primary or secondary amenorrhea between groups.  Age of menarche and contraceptive use patterns were also similar between groups. Among women who did not use contraceptives, most reported regular menses in the year prior to enrolment. |
| Guest et al. 2005[69] | Investigate the associations between high cognitive dietary restraint (CDR) scores and subtle MI, increased cortisol, and lower BMD, increased risk for stress fractures among active women. | Endurance running | There were no significant differences in age, physical, and menstrual cycle characteristics (including the proportion using oral contraceptives), or BMI values between the injured and non-injured groups. |
| Heather et al. 2021[70] | The aim of this study was to quantify the health status of elite female athletes, and understand sociocultural factors influencing that status. | Track and field; Endurance cycling; Rugby; Rowing, field hockey | Stress fractures and iron deficiency were common and associated with oligomenorrhoea/amenorrhea. |
| Henriksson et al. 2000[71] | This study investigated whether menstrual dysfunction was related to MSK injuries among Swedish middle/long distance runners. | Track and field; Endurance running | Women athletes with menstrual dysfunction were found to have had a longer interruption of training due to MSK injuries than those with regular cycles. Athletes using OCs had the highest BMI values, but did not differ in other respects from the other groups (i.e. regular menstruating group and menstrual dysfunction group). |
| Kelsey et al. 2007[72] | To identify risk factors for stress fracture among young female distance runners. | Endurance running | Risk factors for stress fracture among young female runners include previous stress fractures, lower bone mass, and, although not statistically significant in this study, MI. |
| Leinberry et al.1992[73] | To report a stress fracture in the subtrochanteric area of the hip in an amenorrheic runner | Endurance running | Amenorrheic runner, who used OCP for two years sustained a subtrochanteric stress fracture of the hip. |
| Lloyd et al. 1986[74] | To evaluate the effect of menstrual status upon MSK injuries in women athletes. | Endurance running | Stress fractures and fractures were associated with menstrual history. Any injury was associated with no contraceptive use and absent/irregular menstrual status. |
| Micklesfield et al. 2007[75] | To investigate factors associated with menstrual dysfunction, self-reported bone stress injuries and energy balance in women runners. | Endurance running | There was no difference in age, weight, BMI or menstrual function between the runners who did, and did not, report a bone stress injury. The results did not change significantly when the 25% of women using OCPs were excluded. |
| Movaseghi et al. 2012[76] | To evaluate clinical manifestations of the female athlete triad among some elite Iranian athletes. | Track and field; Endurance running; Endurance cycling; Basketball; Football/Soccer; Rugby; All sporting codes in 33 federations (Combat sports, rowing, swimming, Climbing sports, golf, volleyball, bat and ball sports) | Four athletes (33%) had both stress fracture and an ED. One had both stress fracture and low BMD, as well as all three clinical manifestations of the Triad (MI, ED, and low BMD). In general, however, we found no significant association between stress fracture and presence of amenorrhea, oligomenorrhea, age at menarche, or OCP use. |
| Myburgh et al. 1990[77] | To determine whether bone density and other risk factors for osteoporosis associated with stress fractures in athletes | Track and field; Endurance running; Aerobics | Current and past MI, and OCP use was associated with injuries. |
| Prouteau et al. 2004[78] | To identify specific bone characteristics of stress fracture (SF) cases in sportswomen. | Track and field; Triathlon; Dancing; Basketball; Tennis, judo, kick-boxing, golf, step classes | Total duration of amenorrhea in months was also more important in the SF group compared with the C group: 6·7(± 10·7) and 1·2 (± 4·1), respectively (P < 0·05). Use of the contraceptive pill did not differ between groups: SF (n = 10) 79·5 ± 73·8 months C (n = 9) 48·5 ± 46·2 months. These results suggest that the fractal parameter and the BMI at birth may be able to identify female athletes most at risk for this overuse bone injury, as their low indexes might reflect a greater skeletal sensitivity. |
| Puranda et al. 2022[79] | To characterize reproductive health factors in female Canadian Armed Forces (CAF) members and their relationship with MSK injury. | Military | Female members with menstrual cycles reported as never regular, irregular for a few months, who never had a period, and whose periods stopped while serving presented a greater likelihood of RSI were compared to the ones who had regular menstrual cycles. There were associations between use of hormones for birth control and giving birth while serving and injury risk. |
| Rauh et al. 2006[80] | To examine rates and risk factors for overuse injuries among 824 women. | Military | Women who reported secondary amenorrhea (six or more consecutive missed menses during the past 12 months) demonstrated almost a threefold increase in lower-extremity stress fracture risk. No significant associations were found for menstrual variables or birth control hormone use |
| Rauh et al. 2010[81] | To examine the relationship among disordered eating, menstrual dysfunction, and low bone mineral density (BMD) and MSK injury among girls in high school sports | Multiple unspecified | Oligomenorrhea or Amenorrhoea increased risk of injury. No association with menstrual or OCP. |
| Ravi et al. 2021 | To investigate the prevalence of self-reported restrictive eating, current or past eating disorder, and menstrual dysfunction and their relationships with injuries. | Multiple unspecified | Menstrual dysfunction associated with longer RTP (falls away if adjusted for lots of other things).Athletes using HCs were grouped with athletes with other menstrual dysfunctions. |
| Rudolph et al. 2021[82] | To determine differences in health and physical activity history, bone density, microarchitecture, and strength among female athletes with a history of multiple BSI, athletes with ≤1 BSI, and non-athletes. | Multiple unspecified | Age of menarche and current menstrual status (including HC use) were similar among groups. Women with ≥ 3 BSIs had a higher prevalence of amenorrhea compared to both those with ≤1 BSI (p=0.04) and non-athletes (p=0.03; Table 2). Interestingly, among women with ≥ 3 BSIs who reported a history of amenorrhea, 40% reported current menstrual dysfunction compared to 25% of women with ≤1 BSI, and 0% of non-athletes. |
| Cobb et al. 2007[83] | To determine the effect of OCPs on bone mass and stress fracture incidence in young female distance runners. | Endurance running | We found that randomisation to OC had no effect on BMD or BMC in oligo/amenorrhoeic or eumenorrheic female runners, and it yielded a 43% reduction (not statistically significant) in the rate of stress fractures across menstrual groups. |
| Scheinowitz et al. 2017[84] | To evaluate the effects of health habits (including OC-use, MI and age of menarche) and physical activity before recruitment on the fitness level and the incidence of stress fracture during the 4-month army basic training. | Military | The incidence of SF among contraceptive users who exercised before recruitment was 10% compared with 6.4% among those who did not exercise before recruitment (p > 0.05). Although related MI and age of menarche collected (as stated in methods), results were not reported. |
| Thein-Nissenbaum et al. 2014[85] | To determine the prevalence of DE, MI, and MSK injury (INJ; other than stress fractures) in the high school population in OCP users and non-OCP users. | Track and field; Gymnastics; Cheerleading; Dancing; Basketball; Football/Soccer; Multiple unspecified; Tennis, volleyball, swimming, softball, golf | There was no difference in prevalence of MI or MSK injury rate between OCP users and non-users. |
| von Rosen et al. 2017[86] | To survey menarche, MI and use of OCs in female adolescent athletes in the National Sports High Schools in Sweden. A further aim was to study the associations between current sport injury and MI as well as use of OCPs. | Track and field; Triathlon; American football, bowling, cycling, cross country skiing, downhill skiing, freestyle skiing, golf, handball, orienteering, water skiing, wrestling | Current injury was equally distributed in the OC and the non-OC group  but athletes with MI had fewer sports injuries compared to eumenorrheic women. |
| von Rosen et al. 2020[87] | To study the associations between current sport injury and MI as well as use of OCPs. | Endurance running; Endurance cycling; Skiing, triathlon, orienteering | Eumenorrheic women had more current injuries (p = 0.024)  OC was not looked at in conjunction with injury. |
| Wentz et al. 2012[88] | To compare female runners with and without a history of stress fractures to determine possible predictors of such fractures. | Endurance running; cross country running | When considered as individual risk factors no differences were observed for training factors, prior menstrual history, current menstrual status, or use of OCPs between subjects. In logistic regression, stress-fracture risk was increased by the combination of lower dietary calcium intake, history of MI, lower BMD, longer history of running, and running predominantly on hard ground. |
| Wu et al. 2022[89] | To characterize the prevalence of running-related injuries and factors associated with running-related injuries in middle school runners. | Endurance running | Significant associations with injury for higher age of menarche, lower menses in the past 12 months, history of 3 or more missed periods. |

OCP= Oral contraceptive pill; DE =Disordered eating; MI = Menstrual irregularity; BSI= Bone stress index; HC = hormonal contraceptive; SF = stress fracture; BMI = body mass index; BMD = bone mineral density; ED = eating disorder; MSK = musculoskeletal; RSI = relative strength index; RTP = return to play; BMC = bone mineral content;

| Supplementary Table 2f : Studies investigating the association between more than one theme (menstrual cycle phase, menstrual irregularity) and musculoskeletal injury | | | |
| --- | --- | --- | --- |
| **Author and Year** | **Study Aim** | **Sport/Activity** | **Summary of main findings** |
| Petschnig et al. 1997[90] | Stress fracture in a secondary amenorrhea athlete | Table tennis | Athlete with secondary amenorrhea with stress fracture |
| Vico-Moreno et al. 2022[91] | Evaluate associations between 2D biomechanics during landing and proprioception with ankle and knee injuries of female basketball players, considering their menstruation regularity. | Basketball | Data showed that 16% of players sustained an ankle or knee injury, being more frequent in players with irregular menstruation compared to regulars |

2D = Two dimensional

| Supplementary Table 2g : Studies investigating the association between more than one theme (menstrual cycle phase, Menstrual irregularity, and hormonal contraceptive use) and musculoskeletal injury | | | |
| --- | --- | --- | --- |
| **Author and Year** | **Study Aim** | **Sport/Activity** | **Summary of main findings** |
| Doyle et al. 2004[92] | To document and analyse injuries sustained over a competitive season by an elite squad of female rugby players. | Rugby | Data for injury occurrence during specific MCPs were equivocal with eight injuries reported in the first half (follicular phase), 10 injuries reported in the second half (luteal phase) of the menstrual cycle, and for nine injuries, participants could not recall in which part of the cycle their injury occurred. The injury pattern in those subjects who were OCP users, menstrual cycle of regular length and menstrual cycle of irregular length, was consistent with the overall squad injury breakdown. |
| Johnston et al. 2020[93] | The purpose of the study was to compare physiological measures and running-related factors between women of various ages and running abilities with and without a history of running-related stress fractures. | Endurance running | Female runners with low hip bone mineral density, menstrual changes during peak training, and elevated bone turnover markers may be at increased risk of stress fractures. |
| Kadel et al. 1992[94] | To compare dancers with and without stress fractures, considering menstrual status, menstrual history, use of OCPs, calcium intake and daily hours of training. | Dancing | Prolonged amenorrhoeic intervals and heavy training schedules may predispose ballet dancers to stress fractures. Amenorrhea >6months increased odds of stress fractures. |
| Koenig et al. 2008[95] | To report on prospectively collected data pertaining to injuries of the diaphyseal femur occurring at a National Collegiate Athletic Association Division I university. | Track and field; Field hockey, lacrosse, cross country, crew, track | The higher proportion of injured females in this study, and the histories of menstrual irregularities and disordered eating, raised the concern that the female athlete triad may be a factor. |
| Ruedl et al. 2011[96] | The aim of this study was to investigate the interaction of potential intrinsic and extrinsic risk factors in ACL injured recreational female skiers | Skiing | OCP use (P > 0.05) did not affect the ACL injury risk.  Menstrual cycle showed a significant association (P < 0.03). The risk of sustaining an ACL injury is unadjusted 1.9 fold higher in the preovulatory phase compared to the postovulatory phase. |

ACL=Anterior cruciate ligament; OCP=Oral contraceptive pill; MCP = menstrual cycle phase

**Reference list**

[1] MacMahon C, Parrington L. Not All Athletes Are Equal, But Don’t Call Me an Exerciser: Response to Araujo and Scharhag ^1^. Scand J Med Sci Sports 2017;27:904–6. https://doi.org/10.1111/sms.12864.

[2] Araújo CGS, Scharhag J. Athlete: a working definition for medical and health sciences research. Scand J Med Sci Sports 2016;26:4–7. https://doi.org/10.1111/sms.12632.

[3] Bahr R, Clarsen B, Derman W, Dvorak J, Emery CA, Finch CF, et al. International Olympic Committee Consensus Statement: Methods for Recording and Reporting of Epidemiological Data on Injury and Illness in Sports 2020 (Including the STROBE Extension for Sports Injury and Illness Surveillance (STROBE-SIIS)). Orthopaedic Journal of Sports Medicine 2020;8. https://doi.org/10.1177/2325967120902908.

[4] Adachi N, Nawata K, Maeta M, Kurozawa Y. Relationship of the menstrual cycle phase to anterior cruciate ligament injuries in teenaged female athletes. Arch Orthop Trauma Surg 2008;128:473–8. https://doi.org/10.1007/s00402-007-0461-1.

[5] Bambaeichi E, Rahnama N, Barani A. Sport injuries in different phases of menstrual cycle in female athletes. Journal of Isfahan Medical School 2010;28:143–8.

[6] Beynnon BD, Johnson RJ, Braun S, Sargent M, Bernstein IM, Skelly JM, et al. The relationship between menstrual cycle phase and anterior cruciate ligament injury: a case-control study of recreational alpine skiers. American Journal of Sports Medicine 2006;34:757–64. https://doi.org/10.1177/0363546505282624.

[7] Lago-Fuentes C, Padrón-Cabo A, Fernández-Villarino M, Mecías-Calvo M, Muñoz-Pérez I, García-Pinillos F, et al. Follicular phase of menstrual cycle is related to higher tendency to suffer from severe injuries among elite female futsal players. Physical Therapy in Sport 2021;52:90–6.

[8] Lefevre N, Bohu Y, Klouche S, Lecocq J, Herman S. Anterior cruciate ligament tear during the menstrual cycle in female recreational skiers. Orthop Traumatol Surg Res 2013;99:571–5. https://doi.org/10.1016/j.otsr.2013.02.005.

[9] Martin D, Timmins K, Cowie C, Alty J, Mehta R, Tang A, et al. Injury Incidence Across the Menstrual Cycle in International Footballers. Front Sports Act Living 2021;3. https://doi.org/10.3389/fspor.2021.616999.

[10] Pelkowski JN, DeMatas K, McCoy RC, Ortiguera CJ. Seeing Double: A Case of ACL Tears in Monozygotic Twin Female Athletes Within 48 Hours. Cureus 2020;12. https://doi.org/10.7759/cureus.7244.

[11] Reyes SS, Góme JS, Ponce IG, Moraleda BR. Estudio descriptivo de las lesiones de ligamento cruzado en el fútbol femenino. / Descriptive study of cruciate ligament injuries in female soccer. Retos: Nuevas Perspectivas de Educación Física, Deporte y Recreación 2023;50:172–9.

[12] Sommerfield LM, Harrison CB, Whatman CS, Maulder PS. A prospective study of sport injuries in youth females. Phys Ther Sport 2020;44:24–32. https://doi.org/10.1016/j.ptsp.2020.04.005.

[13] Stijak L, Kadija M, Djulejić V, Aksić M, Petronijević N, Marković B, et al. The influence of sex hormones on anterior cruciate ligament rupture: female study. Knee Surgery, Sports Traumatology, Arthroscopy 2015;23:2742–9. https://doi.org/10.1007/s00167-014-3077-3.

[14] Ackerman KE, Cano Sokoloff N, De Nardo Maffazioli G, Clarke HM, Lee H, Misra M. Fractures in Relation to Menstrual Status and Bone Parameters in Young Athletes. Medicine & Science in Sports & Exercise 2015;47:1577–86. https://doi.org/10.1249/MSS.0000000000000574.

[15] Armstrong III DW, Rue J-PH, Wilckens JH, Frassica FJ. Stress fracture injury in young military men and women. Bone 2004;35:806–16. https://doi.org/10.1016/j.bone.2004.05.014.

[16] Barrack MT, Gibbs JC, De Souza MJ, Williams NI, Nichols JF, Rauh MJ, et al. Higher incidence of bone stress injuries with increasing female athlete triad-related risk factors: A prospective multisite study of exercising girls and women. American Journal of Sports Medicine 2014;42:949–58. https://doi.org/10.1177/0363546513520295.

[17] Beals KA, Manore MM. Disorders of the female athlete triad among collegiate athletes. International Journal of Sport Nutrition & Exercise Metabolism 2002;12:281–93. https://doi.org/10.1123/ijsnem.12.3.281.

[18] Bennell KL, Malcolm SA, Thomas SA, Ebeling PR, McCrory PR, Wark JD, et al. Risk factors for stress fractures in female track-and-field athletes: a retrospective analysis. Clin J Sport Med 1995;5:229–35. https://doi.org/10.1097/00042752-199510000-00004.

[19] Benson JE, Geiger CJ, Eiserman PA, Wardlaw GM. Relationship between nutrient intake, body mass index, menstrual function, and ballet injury. J Am Diet Assoc 1989;89:58–63.

[20] Cooper LA, Joy EA. Osteoporosis in a female cross-country runner with femoral neck stress fracture. Curr Sports Med Rep 2005;4:321–2. https://doi.org/10.1097/01.csmr.0000306293.64954.93.

[21] Constantinou D, Saragas NP, Ferrao PN. Bilateral Navicular Stress Fractures with Nonunion in an Adolescent Middle-Distance Athlete: A Case Report. Current Sports Medicine Reports 2021;20:236–41. https://doi.org/10.1249/JSR.0000000000000837.

[22] Edama M, Inaba H, Hoshino F, Saya Natsui, Maruyama S, Omori G. The relationship between the female athlete triad and injury rates in collegiate female athletes. PeerJ 2021. https://doi.org/10.7717/peerj.11092.

[23] Fryar C, Howell DR, Seehusen CN, Tilley D, Casey E, Sweeney EA. Link Between the Female Athlete Triad and Gymnastics-Related Injury in Retired Collegiate Gymnasts. Clinical Journal of Sport Medicine 2023;33:435–9. https://doi.org/10.1097/JSM.0000000000001148.

[24] Goolsby MA, Barrack MT, Nattiv A. A displaced femoral neck stress fracture in an amenorrheic adolescent female runner. Sports Health 2012;4:352–6. https://doi.org/10.1177/1941738111429929.

[25] Haddad FS, Bann S, Hill RA, Jones DH. Displaced stress fracture of the femoral neck in an active amenorrhoeic adolescent. Br J Sports Med 1997;31:70–2. https://doi.org/10.1136/bjsm.31.1.70.

[26] Heikura IA, Uusitalo ALT, Stellingwerff T, Bergland D, Mero AA, Burke LM. Low Energy Availability Is Difficult to Assess but Outcomes Have Large Impact on Bone Injury Rates in Elite Distance Athletes. Int J Sport Nutr Exerc Metab 2018;28:403–11. https://doi.org/10.1123/ijsnem.2017-0313.

[27] Hutson MJ, O’Donnell E, Petherick E. Incidence of bone stress injury is greater in competitive female distance runners with menstrual disturbances independent of participation in plyometric training. Taylor & Francis Verlag 2021.

[28] Ihalainen JK, Kettunen O, McGawley K, Solli GS, Hackney AC, Mero AA, et al. Body Composition, Energy Availability, Training, and Menstrual Status in Female Runners. International Journal of Sports Physiology & Performance 2021;16:1043–8.

[29] Johnson AW, Weiss CBJ, Stento K, Wheeler DL. Stress fractures of the sacrum. An atypical cause of low back pain in the female athlete. Am J Sports Med 2001;29:498–508. https://doi.org/10.1177/03635465010290042001.

[30] Kandemir N, Slattery M, Ackerman KE, Tulsiani S, Bose A, Singhal V, et al. Bone Parameters in Anorexia Nervosa and Athletic Amenorrhea: Comparison of Two Hypothalamic Amenorrhea States. J Clin Endocrinol Metab 2018;103:2392–402. https://doi.org/10.1210/jc.2018-00338.

[31] Korpelainen R, Orava S, Karpakka J, Siira P, Hulkko A. Risk factors for recurrent stress fractures in athletes. Am J Sports Med 2001;29:304–10. https://doi.org/10.1177/03635465010290030901.

[32] Laframboise MA, Borody C, Stern P. The female athlete triad: a case series and narrative overview. J Can Chiropr Assoc 2013;57:316–26.

[33] Lambert BS, Cain MT, Heimdal T, Harris JD, Jotwani V, Petak S, et al. Physiological Parameters of Bone Health in Elite Ballet Dancers. Med Sci Sports Exerc 2020;52:1668–78. https://doi.org/10.1249/MSS.0000000000002296.

[34] Myer GD, Ford KR, Divine JG, Wall EJ, Kahanov L, Hewett TE. Longitudinal assessment of noncontact anterior cruciate ligament injury risk factors during maturation in a female athlete: A case report. Journal of Athletic Training 2009;44:101–9. https://doi.org/10.4085/1062-6050-44.1.101.

[35] Nose-Ogura S, Yoshino O, Yamada-Nomoto K, Nakamura M, Harada M, Dohi M, et al. Oral contraceptive therapy reduces serum relaxin-2 in elite female athletes. PubMed 2017;43. https://doi.org/10.1111/jog.13226.

[36] Okamoto S, Yuji Arai, Hara K, Tsuzihara T, Kubo T. A displaced stress fracture of the femoral neck inan adolescent female distance runner withfemale athlete triad: A case report. SMARTT: Sports Medicine, Arthroscopy, Rehabilitation, Therapy & Technology 2010;2:6–10.

[37] O’Leary TJ, Perrett C, Coombs CV, Double RL, Keay N, Wardle SL, et al. Menstrual disturbances in British Servicewomen: A cross-sectional observational study of prevalence and risk factors. Front Nutr 2022;9. https://doi.org/10.3389/fnut.2022.984541.

[38] Prather H, Hunt D, McKeon K, Simpson S, Meyer EB, Yemm T, et al. Are Elite Female Soccer Athletes at Risk for Disordered Eating Attitudes, Menstrual Dysfunction, and Stress Fractures? PM R 2016;8:208–13. https://doi.org/10.1016/j.pmrj.2015.07.003.

[39] Steinberg N, Siev-Ner I, Peleg S, Dar G, Masharawi Y, Zeev A, et al. Injuries in female dancers aged 8 to 16 years. Journal of Athletic Training 2013;48:118–23. https://doi.org/10.4085/1062-6050-48.1.06.

[40] Tenforde AS, Sayres LC, McCurdy ML, Collado H, Sainani KL, Fredericson M. Overuse Injuries in High School Runners: Lifetime Prevalence and Prevention Strategies. PM and R 2011;3:125–31. https://doi.org/10.1016/j.pmrj.2010.09.009.

[41] Thein-Nissenbaum JM, Rauh MJ, Carr KE, Loud KJ, McGuine TA. Associations between disordered eating, menstrual dysfunction, and musculoskeletal injury among high school athletes. J Orthop Sports Phys Ther 2011;41:60–9. https://doi.org/10.2519/jospt.2011.3312.

[42] Thein-Nissenbaum JM, Rauh MJ, Carr KE, Loud KJ, McGuine TA. Menstrual Irregularity and Musculoskeletal Injury in Female High School Athletes. Journal of Athletic Training 2012;47:74–82.

[43] Tomten S. Prevalence of menstrual dysfunction in Norwegian long-distance runners participating in the Oslo Marathon games. Scandinavian Journal of Medicine & Science in Sports 1996;6:164–71.

[44] Vajapey S, Matic G, Hartz C, Miller TL. Sacral Stress Fractures: A Rare but Curable Cause of Back Pain in Athletes. Sports Health 2019;11:446–52. https://doi.org/10.1177/1941738119854763.

[45] Voss L, DaSilva M, Trafton PG. Bilateral femoral neck stress fractures in an amenorrheic athlete. Am J Orthop (Belle Mead NJ) 1997;26:789–92.

[46] Wilson JH, Wolman RL. Osteoporosis and fracture complications in an amenorrhoeic athlete. Br J Rheumatol 1994;33:480–1. https://doi.org/10.1093/rheumatology/33.5.480.

[47] Witkoś J, Błażejewski G, Hagner-Derengowska M, Makulec K. The Impact of Competitive Swimming on Menstrual Cycle Disorders and Subsequent Sports Injuries as Related to the Female Athlete Triad and on Premenstrual Syndrome Symptoms. Int J Environ Res Public Health 2022;19. https://doi.org/10.3390/ijerph192315854.

[48] Wolman RL. Association between the menstrual cycle and anterior cruciate ligament in female athletes. Am J Sports Med 1999;27:270–1.

[49] Agel J, Bershadsky B, Arendt EA. Hormonal therapy: ACL and ankle injury. Medicine & Science in Sports & Exercise 2006;38:7–12. https://doi.org/10.1249/01.mss.0000194072.13021.78.

[50] Liederbach M, Dilgen FE, Rose DJ. Incidence of anterior cruciate ligament injuries among elite ballet and modern dancers: a 5-year prospective study. Am J Sports Med 2008;36:1779–88. https://doi.org/10.1177/0363546508323644.

[51] Niyonsenga JD, Phillips JS. Factors associated with injuries among first-division Rwandan female soccer players. Afr Health Sci 2013;13:1021–6. https://doi.org/10.4314/ahs.v13i4.23.

[52] Redinger AL, Baker BS. Oral Contraceptives and Female Rowers’ Skeletal Health. Journal of Strength and Conditioning Research 2023;37:669–77. https://doi.org/10.1519/JSC.0000000000004308.

[53] Arendt EA, Bershadsky B, Agel J. Periodicity of noncontact anterior cruciate ligament injuries during the menstrual cycle. Journal of Gender-Specific Medicine 2002;5:19–26.

[54] Dragoo JL, Castillo TN, Braun HJ, Ridley BA, Kennedy AC, Golish SR. Prospective correlation between serum relaxin concentration and anterior cruciate ligament tears among elite collegiate female athletes. Am J Sports Med 2011;39:2175–80. https://doi.org/10.1177/0363546511413378.

[55] Myklebust G, Maehlum S, Holm I, Bahr R. A prospective cohort study of anterior cruciate ligament injuries in elite Norwegian team handball. Scand J Med Sci Sports 1998;8:149–53. https://doi.org/10.1111/j.1600-0838.1998.tb00185.x.

[56] Moller-Nielsen J, Hammar M. Women’s soccer injuries in relation to the menstrual cycle and oral contraceptive use. / Traumatologie du football chez les femmes, en relation avec le cycle menstruel et l’ utilisation de contraceptifs oraux. Medicine & Science in Sports & Exercise 1989;21:126–9.

[57] Ruedl G, Ploner P, Linortner I, Schranz A, Fink C, Sommersacher R, et al. Are oral contraceptive use and menstrual cycle phase related to anterior cruciate ligament injury risk in female recreational skiers? PubMed 2009;17. https://doi.org/10.1007/s00167-009-0786-0.

[58] Slauterbeck JR, Fuzie SF, Smith MP, Clark RJ, Xu KT, Starch DW, et al. The menstrual cycle, sex hormones, and anterior cruciate ligament injury...including commentary by Kirk SE with author response. Journal of Athletic Training (National Athletic Trainers’ Association) 2002;37:275–80.

[59] Wojtys EM, Huston LJ, Lindenfeld TN, Hewett TE, Greenfield ML. Association between the menstrual cycle and anterior cruciate ligament injuries in female athletes. PubMed 1998;26. https://doi.org/10.1177/03635465980260050301.

[60] Wojtys EM, Huston LJ, Boynton MD, Spindler KP, Lindenfeld TN. The effect of the menstrual cycle on anterior cruciate ligament injuries in women as determined by hormone levels. American Journal of Sports Medicine 2002;30:182–8. https://doi.org/10.1177/03635465020300020601.

[61] Barrow GW, Saha S. Menstrual irregularity and stress fractures in collegiate female distance runners. Am J Sports Med 1988;16:209–16. https://doi.org/10.1177/036354658801600302.

[62] Bennell KL, Malcolm SA, Thomas SA, Reid SJ, Brukner PD, Ebeling PR, et al. Risk factors for stress fractures in track and field athletes. A twelve-month prospective study. Am J Sports Med 1996;24:810–8. https://doi.org/10.1177/036354659602400617.

[63] Cheng J, Santiago KA, Abutalib Z, Temme KE, Hulme A, Goolsby MA, et al. Menstrual Irregularity, Hormonal Contraceptive Use, and Bone Stress Injuries in Collegiate Female Athletes in the United States. PM&amp;R 2021;13:1207–15. https://doi.org/10.1002/pmrj.12539.

[64] Cline AD, Jansen GR, Melby CL. Stress fractures in female army recruits: implications of bone density, calcium intake, and exercise. J Am Coll Nutr 1998;17:128–35. https://doi.org/10.1080/07315724.1998.10718738.

[65] Duckham RL, Brooke-Wavell K, Summers GD, Cameron N, Peirce N. Stress fracture injury in female endurance athletes in the United Kingdom: A 12-month prospective study. Scand J Med Sci Sports 2015;25:854–9. https://doi.org/10.1111/sms.12453.

[66] Dugowson CE, Drinkwater BL, Clark JM. Nontraumatic femur fracture in an oligomenorrheic athlete. Med Sci Sports Exerc 1991;23:1323–5.

[67] Eller DJ, Katz DS, Bergman AG, Fredericson M, Beaulieu CF. Sacral stress fractures in long-distance runners. Clinical Journal of Sport Medicine 1997;7:222–5. https://doi.org/10.1097/00042752-199707000-00014.

[68] Gehman S, Ackerman KE, Caksa S, Rudolph SE, Hughes JM, Garrahan M, et al. Restrictive Eating and Prior Low-Energy Fractures Are Associated With History of Multiple Bone Stress Injuries. International Journal of Sport Nutrition and Exercise Metabolism 2022;32:325–33. https://doi.org/10.1123/ijsnem.2021-0323.

[69] Guest NS, Barr SI. Cognitive dietary restraint is associated with stress fractures in women runners. Int J Sport Nutr Exerc Metab 2005;15:147–59. https://doi.org/10.1123/ijsnem.15.2.147.

[70] Heather AK, Thorpe H, Ogilvie M, Sims ST, Beable S, Milsom S, et al. Biological and Socio-Cultural Factors Have the Potential to Influence the Health and Performance of Elite Female Athletes: A Cross Sectional Survey of 219 Elite Female Athletes in Aotearoa New Zealand. Front Sports Act Living 2021;3. https://doi.org/10.3389/fspor.2021.601420.

[71] Beckvid Henriksson G, Schnell C, Lindén Hirschberg A. Women endurance runners with menstrual dysfunction have prolonged interruption of training due to injury. Gynecologic and Obstetric Investigation 2000;49:41–6. https://doi.org/10.1159/000010211.

[72] Kelsey JL, Bachrach LK, Procter-Gray E, Nieves J, Greendale GA, Sowers M, et al. Risk factors for stress fracture among young female cross-country runners. Med Sci Sports Exerc 2007;39:1457–63. https://doi.org/10.1249/mss.0b013e318074e54b.

[73] Leinberry CF, McShane RB, Stewart WGJ, Hume EL. A displaced subtrochanteric stress fracture in a young amenorrheic athlete. Am J Sports Med 1992;20:485–7. https://doi.org/10.1177/036354659202000425.

[74] Lloyd T, Triantafyllou SJ, Baker ER, Houts PS, Whiteside JA, Kalenak A, et al. Women athletes with menstrual irregularity have increased musculoskeletal injuries. Med Sci Sports Exerc 1986;18:374–9.

[75] Micklesfield LK, Hugo J, Johnson C, Noakes TD, Lambert EV. Factors associated with menstrual dysfunction and self-reported bone stress injuries in female runners in the ultra- and half-marathons of the Two Oceans. Br J Sports Med 2007;41:679–83. https://doi.org/10.1136/bjsm.2007.037077.

[76] Movaseghi S, Dadgostar H, Dahaghin S, Chimeh N, Alenabi T, Dadgostar E, et al. Clinical manifestations of the female athlete triad among some Iranian athletes. Medicine and Science in Sports and Exercise 2012;44:958–65. https://doi.org/10.1249/MSS.0b013e31823bd057.

[77] Myburgh KH, Hutchins J, Fataar AB, Hough SF, Noakes TD. Low bone density is an etiologic factor for stress fractures in athletes. Ann Intern Med 1990;113:754–9. https://doi.org/10.7326/0003-4819-113-10-754.

[78] Prouteau S, Ducher G, Nanyan P, Lemineur G, Benhamou L, Courteix D. Fractal analysis of bone texture: a screening tool for stress fracture risk? Eur J Clin Invest 2004;34:137–42. https://doi.org/10.1111/j.1365-2362.2004.01300.x.

[79] Puranda JL, Silva DF da, Edwards CM, Nagpal TS, Souza SS, Semeniuk K, et al. Association Between Reproductive Health Factors and Musculoskeletal Injuries in Female Canadian Armed Forces Members. Journal of Women’s Health (2002) 2022. https://doi.org/10.1089/jwh.2021.0647.

[80] Rauh MJ, Macera CA, Trone DW, Shaffer RA, Brodine SK. Epidemiology of Stress Fracture and Lower-Extremity Overuse Injury in Female Recruits. Medicine & Science in Sports & Exercise 2006;38:1571–7.

[81] Rauh MJ, Nichols JF, Barrack MT. Relationships among injury and disordered eating, menstrual dysfunction, and low bone mineral density in high school athletes: a prospective study. J Athl Train 2010;45:243–52. https://doi.org/10.4085/1062-6050-45.3.243.

[82] Rudolph SE, Caksa S, Gehman S, Garrahan M, Hughes JM, Tenforde AS, et al. Physical Activity, Menstrual History, and Bone Microarchitecture in Female Athletes with Multiple Bone Stress Injuries. Med Sci Sports Exerc 2021;53:2182–9. https://doi.org/10.1249/MSS.0000000000002676.

[83] Cobb KL, Bachrach LK, Sowers M, Nieves J, Greendale GA, Kent KK, et al. The effect of oral contraceptives on bone mass and stress fractures in female runners. Med Sci Sports Exerc 2007;39:1464–73. https://doi.org/10.1249/mss.0b013e318074e532.

[84] Scheinowitz M, Yanovich R, Sharvit N, Arnon M, Moran DS. Effect of cardiovascular and muscular endurance is not associated with stress fracture incidence in female military recruits: a 12-month follow up study. J Basic Clin Physiol Pharmacol 2017;28:219–24. https://doi.org/10.1515/jbcpp-2015-0098.

[85] Thein-Nissenbaum JM, Carr KE, Hetzel S, Dennison E. Disordered Eating, Menstrual Irregularity, and Musculoskeletal Injury in High School Athletes. Sports Health 2014;6:313–20. https://doi.org/10.1177/1941738113498852.

[86] von Rosen P, Heijne A, Frohm A, Fridén C. Menstrual irregularity and use of oral contraceptives in female adolescent athletes in Swedish National Sports High Schools. International Journal of Adolescent Medicine and Health 2017;32. https://doi.org/10.1515/ijamh-2017-0113.

[87] Von Rosen P, Heijne A, Frohm A, Fridén C. Menstrual irregularity and use of oral contraceptives in female adolescent athletes in Swedish National Sports High Schools. International Journal of Adolescent Medicine and Health 2020;32. https://doi.org/10.1515/ijamh-2017-0113.

[88] Wentz L, Pei-Yang Liu, Ilich JZ, Haymes EM. Dietary and Training Predictors of Stress Fractures in Female Runners. International Journal of Sport Nutrition & Exercise Metabolism 2012;22:374–82.

[89] Wu AC, Rauh MJ, DeLuca S, Lewis M, Ackerman KE, Barrack MT, et al. Running-related injuries in middle school cross-country runners: Prevalence and characteristics of common injuries. PM R 2022;14:793–801. https://doi.org/10.1002/pmrj.12649.

[90] Petschnig R, Wurnig C, Rosen A, Baron R. Stress fracture of the ulna in a female table tennis tournament player. J Sports Med Phys Fitness 1997;37:225–7.

[91] Vico-Moreno E, Sastre-Munar A, Fernández-Domínguez JC, Romero-Franco N. Motor Control and Regularity of Menstrual Cycle in Ankle and Knee Injuries of Female Basketball Players: A Cohort Study. Int J Environ Res Public Health 2022;19. https://doi.org/10.3390/ijerph192114357.

[92] Doyle C, George K. Injuries associated with elite participation in women’s rugby over a competitive season: An initial investigation. Physical Therapy in Sport 2004;5:44–50. https://doi.org/10.1016/j.ptsp.2003.11.001.

[93] Johnston TE, Dempsey C, Gilman F, Tomlinson R, Jacketti A-K, Close J. Physiological Factors of Female Runners With and Without Stress Fracture Histories: A Pilot Study. Sports Health 2020;12:334–40. https://doi.org/10.1177/1941738120919331.

[94] Kadel NJ, Teitz CC, Kronmal RA. Stress fractures in ballet dancers. Am J Sports Med 1992;20:445–9. https://doi.org/10.1177/036354659202000414.

[95] Koenig SJ, Toth AP, Bosco JA. Stress fractures and stress reactions of the diaphyseal femur in collegiate athletes: an analysis of 25 cases. American Journal of Orthopedics (Belle Mead, NJ) 2008;37:476–80.

[96] Ruedl G, Ploner P, Linortner I, Schranz A, Fink C, Patterson C, et al. Interaction of potential intrinsic and extrinsic risk factors in ACL injured recreational female skiers. Int J Sports Med 2011;32:618–22. https://doi.org/10.1055/s-0031-1275355.
